# FEDS: a Novel Fluorescence-Based High-Throughput Method for Measuring DNA Supercoiling *In Vivo*

**DOI:** 10.1128/mBio.01053-20

**Published:** 2020-07-28

**Authors:** Alexandre Duprey, Eduardo A. Groisman

**Affiliations:** a Department of Microbial Pathogenesis, Yale School of Medicine, New Haven, Connecticut, USA; b Yale Microbial Sciences Institute, West Haven, Connecticut, USA; The Ohio State University

**Keywords:** DNA gyrase, DNA topology, feedback loop, gene transcription

## Abstract

DNA supercoiling (DS) is essential for life because it controls critical processes, including transcription, replication, and recombination. Current methods to measure DNA supercoiling *in vivo* are laborious and unable to examine single cells. Here, we report a method for high-throughput measurement of bacterial DNA supercoiling *in vivo*. Fluorescent evaluation of DNA supercoiling (FEDS) utilizes a plasmid harboring the gene for a green fluorescent protein transcribed by a discovered promoter that responds exclusively to DNA supercoiling and the gene for a red fluorescent protein transcribed by a constitutive promoter as the internal standard. Using FEDS, we uncovered single-cell heterogeneity in DNA supercoiling and established that, surprisingly, population-level decreases in DNA supercoiling result from a low-mean/high-variance DNA supercoiling subpopulation rather than from a homogeneous shift in supercoiling of the whole population. In addition, we identified a regulatory loop in which a gene that decreases DNA supercoiling is transcriptionally repressed when DNA supercoiling increases.

## Abstract (precis)

DNA represents the chemical support of genetic information in all forms of life. In addition to its linear sequence of nucleotides, it bears critical information in its structure. This information, called DNA supercoiling, is central to all fundamental DNA processes, such as transcription and replication, and defines cellular physiology. Unlike reading of a nucleotide sequence, DNA supercoiling determinations have been laborious. We have now developed a method for rapid measurement of DNA supercoiling and established its utility by identifying a novel regulator of DNA supercoiling in the bacterium Salmonella enterica as well as behaviors that could not have been discovered with current methods.

## INTRODUCTION

DNA represents the chemical support for the genetic material. Known as DNA supercoiling (DS), the twisting and writhing of the DNA double helix enables compaction of the large DNA molecule into the limited cellular space (1). Essential cellular processes, such as transcription, replication, and recombination, are strongly affected by DNA supercoiling (2–7). For example, DNA wrapped by histones tends to be silent (8). The tight control of DNA supercoiling is critical for cell function; for example, the bacterium Escherichia coli loses viability upon a 15% decrease in DNA supercoiling (9), human immunodeficiency virus DNA cannot integrate into sufficiently relaxed DNA (3), and DNA cleavage by CRISPR Cas12a is favored when DNA is negatively supercoiled (10).

On average, DNA tends to be negatively supercoiled in bacteria. However, DNA supercoiling varies along the bacterial chromosome, with regions being more or less supercoiled under a given set of growth conditions (5, 9) or responding differently to pharmacological perturbation of the average supercoiling density (11). Therefore, bacterial behaviors reflect not only global DNA supercoiling but also local supercoil density.

Current methods for measuring DNA supercoiling *in vivo* are slow and laborious. The reference method involves extracting a reporter plasmid from cells and running it on an agarose/chloroquine gel to resolve the different DNA conformers (12). The data obtained by this method reflect the average negative DNA supercoiling of the chromosome, but the method is incapable of visualizing local DNA supercoiling. The reference method cannot be used in single cells, which is problematic given that certain biological phenomena are revealed only by investigating the behavior of single cells (13), and is infeasible for large-scale screens. Psoralen cross-linking (14) and recombination-based strategies (5) improved some aspects of the original method, but not the low throughput. Although recent approaches designed to examine topoisomerase activity *in vitro* are more efficient than those used in the past (15–17), they are not applicable in living cells.

Here, we report the development of fluorescent evaluation of DNA supercoiling (FEDS), a method to measure DNA supercoiling *in vivo* that is fast, easy to use, and compatible with single-cell approaches, such as microscopy and flow cytometry. FEDS relies on a plasmid with two promoters: (i) a newly discovered promoter that is exclusively regulated by DNA supercoiling and drives transcription of the gene for a green fluorescent protein and (ii) a bona fide constitutive promoter that drives transcription of the gene for a red fluorescent protein. We validated FEDS by demonstrating that it faithfully reports *in vivo* negative DNA supercoiling in two bacterial species with different basal DNA supercoiling characteristics. We established that the bacterium Salmonella enterica serovar Typhimurium exhibits single-cell heterogeneity in DNA supercoiling and that conditions that trigger population-level decreases in DNA supercoiling result from a low-mean/high-variance supercoiling subpopulation (rather than from a homogeneous shift in the mean supercoiling of the whole population). In addition, we discovered a regulatory loop in which DNA supercoiling represses transcription of a gene that reduces DNA supercoiling. FEDS reveals genetic determinants and physiological signals governing DNA supercoiling in living cells.

## RESULTS

### Design principles for the construction of a reporter of *in vivo* DNA supercoiling.

We sought to construct a reporter of DNA supercoiling having the following desirable qualities: high sensitivity and specificity, ease of detection and quantification, and minimal impact on cell physiology (18). Current DNA supercoiling reporters satisfy only the sensitivity and specificity aspects. Thus, we designed a method for measuring *in vivo* DNA supercoiling that satisfies all five criteria by exploiting easy-to-use fluorescent proteins.

The FEDS method relies on four genetic elements located in a multicopy plasmid (designated “pSupR” for “supercoiling reporter”). These four elements are (i) a promoterless gene specifying a green fluorescent protein (*gfpmut3*; https://www.fpbase.org/protein/gfpmut3/) directly controlled by (ii) a promoter exclusively regulated by DNA supercoiling and an internal standard that includes (iii) a promoterless version of a gene specifying a red fluorescent protein (*tdtomato*, https://www.fpbase.org/protein/tdtomato/) controlled by (iv) a constitutive promoter. The strength of the two promoters should be high enough for the fluorescence output to be detectable, but not so high that it disrupts cellular physiology. The internal standard corrects for variables that can affect the fluorescence output, such as the amount of ATP available for protein synthesis (19), the plasmid copy number (20), asymmetric plasmid segregation at cell division (21), and plasmid loss. By measuring both green and red fluorescence, FEDS enables comparisons across different physiological states, ensuring sensitivity and specificity.

**FIG 1:**
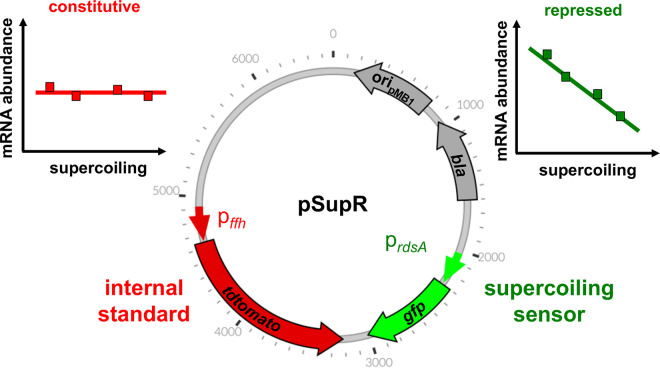
Map of pSupR, a supercoiling reporter plasmid for enterobacteria. It includes one constitutive promoter (red, left side) and one promoter exclusively repressed by DNA supercoiling (green, right side), each transcribing either of two genes coding for different fluorescent proteins, the origin of replication from plasmid pMB1 and the *bla* gene conferring resistance to ampicillin.

Plasmid pSupR harbors the *gfp* gene, the *tdtomato* gene, a selectable marker, and the origin of replication of plasmid pMB1 (Fig. 1), which is present in the commonly used vector pBR322 (22) and operates in multiple enterobacterial species. The two fluorescent protein-encoding genes are placed convergently, which ensures both maximum distance between the corresponding promoter regions and minimal interference between promoters (23). The *gfp* and *tdtomato* genes are transcribed from DNA supercoiling-responsive and constitutive promoters, respectively. We did not include transcriptional terminators at the end of the *gfp* and *tdtomato* genes because, as demonstrated below, pSupR faithfully reports *in vivo* DNA supercoiling behavior obtained with the classical agarose/chloroquine gel method (24). In sum, pSupR allows direct and immediate measurement of DNA supercoiling using common equipment, thereby ensuring ease of detection and quantification.

### A coupled DNA supercoiling/transcriptome sequencing (RNA-seq) experiment identifies genes regulated by DNA supercoiling and genes impervious to DNA supercoiling.

To identify candidates for a constitutive promoter and for a promoter whose activity varies in a predictable fashion as a function of DNA supercoiling, we measured both negative DNA supercoiling and gene expression in the same cultures of wild-type S. enterica serovar Typhimurium strain 14028s (or of isogenic mutants where appropriate). We used 11 different conditions known to alter DNA supercoiling, including growth in defined media, complex media, exposure to abiotic stresses, and changes in the concentrations of specific ions (Table 1). These conditions alter a wide variety of cellular functions in addition to DNA supercoiling, allowing the identification of robust constitutive and supercoiling-dependent genes. For each condition, we determined negative DNA supercoiling data using the classical agarose/chloroquine gel method (24) on extracted plasmid DNA and genome-wide mRNA abundance data using RNA-seq. Then, we matched a negative DNA supercoiling value for the mRNA abundance of each gene in the *Salmonella* genome.

**TABLE 1:** Conditions used to examine RNA abundance and DNA supercoiling in the same bacterial cultures

Genotype of *Salmonella*	Medium	DNA supercoiling (RSU)
Wild type	HH + 150 μg/ml novobiocin, 10 min	−3.19
Wild type	HH + 25 μg/ml novobiocin	−2.15
Wild type	HH + 2 mM H_2_O_2_, 10 min	−1.41
Wild type	HH	0.00
*fis*	HH	0.54
Wild type	HH800	1.00
Wild type	HH800 + 300 mM NaCl	1.46
Wild type	HH + 100 μM FeSO_4_	1.64
*pmrA*	HH800	3.14
*speE-oat*	HH800	3.99
Wild type	LB	5.31

We determined that 11 conditions were sufficient to achieve a true-positive rate of 63% for the detection of genes regulated exclusively by DNA supercoiling (see Fig. S1 in the supplemental material), which gave us a good chance of isolating promoters of interest at later stages. Improving the true-positive rate would have been prohibitively difficult and expensive as it would have taken 18 conditions to reach a true-positive rate of 83% and 30 conditions for a true-positive rate of 95% (Fig. S1). Below, we discuss the identification of a constitutive promoter and a DNA supercoiling-regulated promoter.

10.1128/mBio.01053-20.2 FIG S1 Estimation of the true-positive rate given by the number of conditions used in the RNA-seq experiment. Data from *n* conditions (*x* axis) of the 11-condition RNA-seq experiment were randomly shuffled, and the number of genes passing the cutoff score of 2.9 was computed. The average number of genes passing the cutoff was then fitted to a power law that asymptotically converges to the number of expected true positives (5 in this case). Download FIG S1, PDF file, 0.05 MB. Copyright © 2020 Duprey and Groisman. 2020 Duprey and Groisman. This content is distributed under the terms of the Creative Commons Attribution 4.0 International license.

### The *ffh* promoter is constitutive and serves as an internal standard.

By definition, constitutive promoters are those that are active to the same degree no matter the growth condition (25). To identify constitutive promoters from our data set, we applied the following rationale: the mRNA amounts of a constitutively expressed gene should have little variation across all 11 conditions. Consequently, we ordered genes according to the relative differences between their maximum and minimum expression values. We chose promoters associated with genes that (i) are first in an operon, (ii) are expressed at a reasonable level (>100 fragments per kilobase per million [FPKM]), (iii) display <40% variation between maximum and minimum expression values, and (iv) have no known transcriptional or posttranscriptional regulation in *Salmonella* or E. coli. The promoters of the *imp* and *ffh* genes satisfied these criteria and were investigated further.

To experimentally validate the candidate promoters, we devised a test to verify that a promoter is constitutive. We reasoned that if two promoters are constitutive, their expression should be exactly correlated. To avoid selecting promoters that display the same behavior because they are controlled by the same transcription factor, the two promoters must also be phylogenetically unrelated (i.e., not issued from a duplication event). Therefore, in a set of *n* = >2 potentially constitutive promoters that contained at least two constitutive promoters, the constitutive promoters would correlate linearly with each other, whereas the nonconstitutive promoters would deviate from a linear correlation. This test, designated the correlation clustering test (CCT), is independent of the reporter and method used to measure their activity.

We examined the behavior of four promoters across multiple conditions by measuring the fluorescence of *Salmonella* harboring a plasmid with a promoter fusion to the *tdtomato* promoterless gene. The *imp* and *ffh* promoters were chosen from the RNA-seq data set analysis; J23100 is a synthetic constitutive promoter (http://parts.igem.org/Part:BBa_J23100), and J23119 is a stronger derivative of J23100. The *imp*, *ffh*, and J23100 promoters, which are phylogenetically unrelated, correlated best, with correlation coefficient (*R*^2^) values of >0.9 (Table 2). By contrast, J23119 correlated less well with the other promoters, including its parent, J23100 (*R*^2^ = 0.7). Therefore, we verified that the J23100, *imp*, and *ffh* promoters are constitutive.

**TABLE 2:** Correlation coefficients between potentially constitutive promoters

Promoter	*R*^2^ value^*a*^			
	J23119	J23100	*imp*	*ffh*
J23119	1			
J23100	0.693	1		
*imp*	0.569	0.906	1	
*ffh*	0.622	0.928	0.924	1

a *R*^2^, correlation coefficient.

To build the DNA supercoiling reporter plasmid, we chose the *ffh* promoter because it was the weakest of the three and yet still produced a fluorescence signal 10-fold higher than background even right after inoculation. That the *ffh* gene is an experimentally validated quantitative reverse transcription-PCR (qRT-PCR) standard in *Erwinia* (26) provides independent support for the notion that *ffh* is transcribed from a constitutive promoter.

### The *ydeJ* promoter responds exclusively to DNA supercoiling.

**FIG 2:**
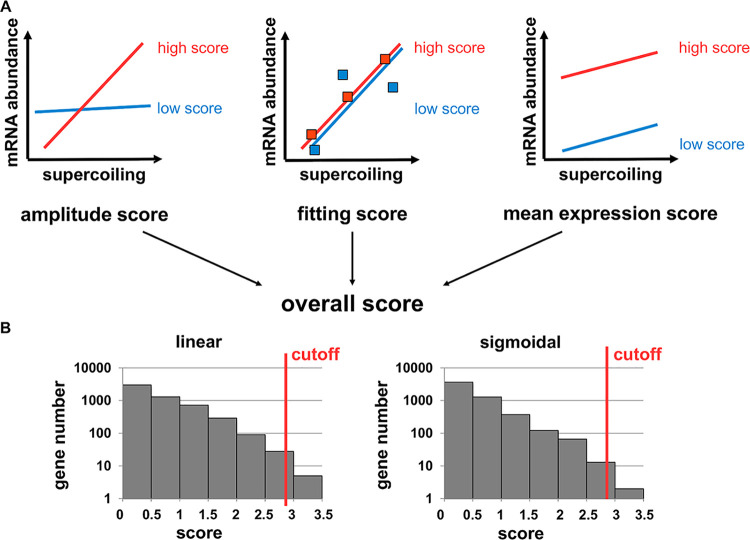
A scoring system to identify promoters responding exclusively to DNA supercoiling. (A) Genes in the S. enterica serovar Typhimurium 14028s genome were ranked according to their expression properties (as measured by RNA-seq). The amplitude score (maximum of 3 points) and fitting score (maximum of 1 point) rewarded genes that vary strongly and predictably with DNA supercoiling, respectively, and the mean expression score (maximum of 1 point) penalized genes whose expression is weak. The overall score (i.e., the sum of the three individual scores) was used to rank genes that were desirable as supercoiling reporters. (B) Distribution of scores depending on the regression type used. A cutoff value of 2.9 was chosen empirically.

To identify DNA supercoiling-responsive genes from the data set, we developed a scoring system that satisfies our rationale for an effective DNA supercoiling reporter (Fig. 2A). The system is based on three scores processed into an overall score. (i) The amplitude score represents the difference between the minimum and maximum gene expression values under the investigated conditions. (ii) The fitting score reflects the dispersion of individual points compared to the regression. (iii) The mean expression score corresponds to the mean mRNA amount.

To cover the widest range of gene expression patterns possible, we used both linear and logistic regressions to analyze the data. Based on the histogram of score distributions (Fig. 2B), a cutoff score of 2.9 was empirically chosen. We considered for further testing the 11 genes scoring better than this cutoff in regression analyses (see Data Set S2 in the supplemental material). Three additional genes that had an excellent fitting score but did not make it past the cutoff due to a low mean expression score (*ydeJ*, *rbfA*, and *STM14_2665*) (Data Set S2) were added to the 11 genes chosen.

**FIG 3:**
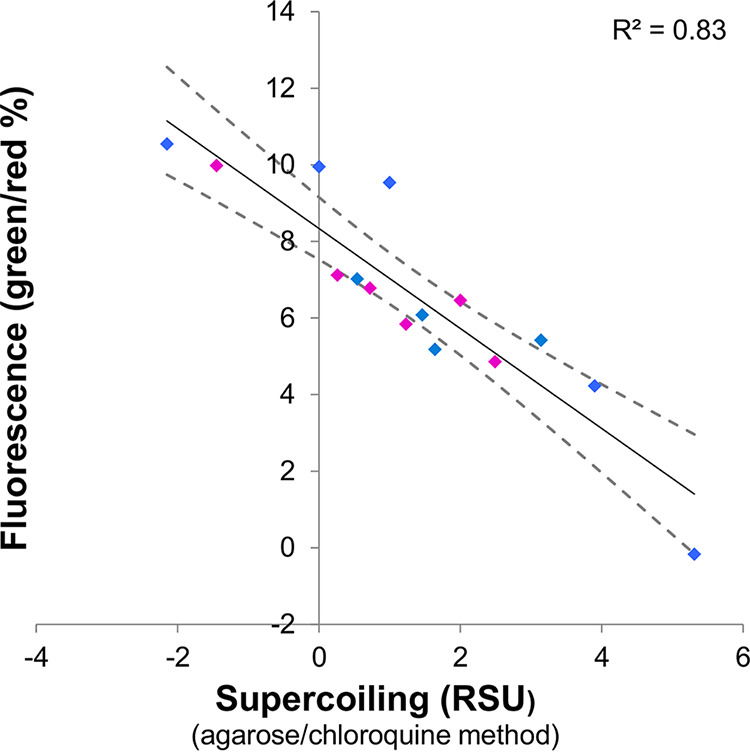
Expression of the *ydeJ* gene is inversely correlated with DNA supercoiling. S. enterica serovar Typhimurium wild-type strain 14028s or isogenic mutants bearing plasmid pSupR or pJV were grown in a variety of media. Strains bearing pJV were used to measure DNA supercoiling by the agarose/chloroquine gel method. Strains bearing pSupR were used to measure the fluorescence ratios. Blue data points indicate conditions corresponding to the 11 conditions used in the RNA-seq experiment; red data points indicate conditions that alter DNA supercoiling but that were not used in the RNA-seq experiment. A full description of the study conditions is available in Table S1B.

We cloned the promoter regions of 8 of the 14 genes in front of a promoterless *gfp* gene. All eight promoters were screened using external normalization to the fluorescence of a separate plasmid harboring the *ffh* promoter driving transcription of the promoterless *tdtomato* gene (pFITL, see Table S1A in the supplemental material). Promoters varying with negative DNA supercoiling as expected were further validated with internal normalization to *ffh* (i.e., with the promoter fusions to *gfp* and the p*ffh-tdtomato* on the same plasmid). Of the eight promoters, two conferred no detectable expression, four conferred expression behavior inconsistent with that observed in the RNA-seq analysis of the corresponding gene, and one conferred fluorescence in a consistent trend but produced data that were too imprecise for it to be used as a reporter. The remaining promoter, corresponding to the *ydeJ* gene, was strongly inversely correlated with negative DNA supercoiling across all investigated conditions (*R*^2^ = 0.82) (Fig. 3).

10.1128/mBio.01053-20.8 TABLE S1 (A) Summary of materials used in this study. (B) Description of the 15 conditions used to validate pSupR. Download Table S1, DOCX file, 0.2 MB. Copyright © 2020 Duprey and Groisman. 2020 Duprey and Groisman. This content is distributed under the terms of the Creative Commons Attribution 4.0 International license.

Control experiments were carried out with wild-type *Salmonella* harboring plasmid pFLTL, which is similar to pSupR except that the constitutive *ffh* promoter controls the *gfp* gene instead of the *ydeJ* promoter. The green and red fluorescence from pFLTL-containing bacteria showed no relationship with negative DNA supercoiling because (i) the slope of the fit was nearly equal to zero (0.28 ± 0.10) and (ii) the correlation between the red to green fluorescence ratios in pFLTL- and pSupR-containing bacteria was minimal (*R*^2^ = 0.15) (Fig. S2). Therefore, the fluorescence of pSupR-containing bacteria faithfully reflected negative DNA supercoiling, as opposed to being an intrinsic property of the fluorescent proteins.

10.1128/mBio.01053-20.3 FIG S2 Comparison of the green fluorescence to red fluorescence ratios in wild-type *Salmonella* carrying plasmid pFLTL or plasmid pSupR. Data represent ratios of green fluorescence to red fluorescence produced by wild-type *S.* Typhimurium (14028s) harboring plasmid pFLTL, in which the constitutive *ffh* promoter drives transcription of a promoterless *gfp* gene and (separately) of a promoterless *dtomato* gene, or plasmid pSupR, in which the supercoiling-responsive *ydeJ* promoter drives transcription of a promoterless *gfp* gene and the constitutive *ffh* promoter drives transcription of a promoterless *dtomato* gene. Bacteria were grown in the following media: HH, HH800, HH800 pH 4.6, and HH plus novobiocin (25 μg/ml). Data are represented as individual points (gray squares), fitted to a linear model (black line), and 95% confidence intervals for the fit (dotted lines). Download FIG S2, PDF file, 0.1 MB. Copyright © 2020 Duprey and Groisman. 2020 Duprey and Groisman. This content is distributed under the terms of the Creative Commons Attribution 4.0 International license.

**FIG 4:**
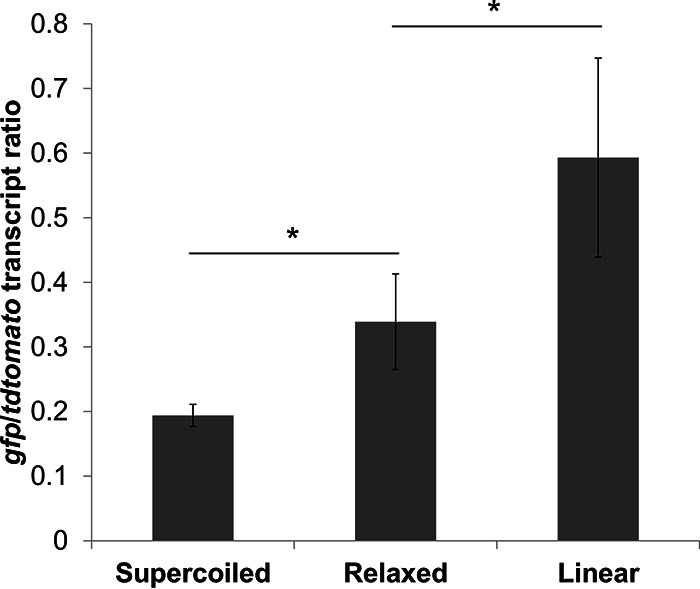
pSupR responds to DNA supercoiling *in vitro*. *In vitro* transcription was performed using supercoiled pSupR, relaxed pSupR (treated with topoisomerase I), or linear pSupR (linearized by restriction digestion). Transcripts were quantified by qPCR, and the ratio of *gfp* transcripts to *tdtomato* transcripts is presented. *, *P* < 0.05 (Student's *t* test, *n* = 3).

The supercoiling sensitivity of pSupR was also conserved *in vitro*, as transcription from the *ydeJ* promoter relative to the *ffh* promoter on purified pSupR depended on supercoiling (Fig. 4). That is, the *gfp*/*tdtomato* transcript ratio was minimal on supercoiled DNA, was increased on relaxed DNA, and was maximal on linear DNA (Fig. 4). This suggests that the supercoiling sensitivity of pSupR is intrinsic and does not depend on any other external factor.

In sum, plasmid pSupR bears the supercoiling-sensitive *ydeJ* promoter controlling Gfp production and the constitutive *ffh* promoter controlling tdTomato production, both originating from the *S.* Typhimurium strain 14028s genome. In cells carrying pSupR, negative DNA supercoiling is linearly anticorrelated to the ratio of green to red fluorescence, which is the basis for the FEDS method.

### FEDS unveils a new regulator of DNA supercoiling.

Bacterial regulatory networks often comprise feedback loops (27, 28). Thus, we wondered whether the *ydeJ* (STM14_1830) gene product regulates DNA supercoiling given that the activity of the *ydeJ* promoter decreases as negative DNA supercoiling increases (Fig. 3). To test this possibility, we used both the novel FEDS method (Fig. 1) and the classical agarose/chloroquine method (12) to determine the DNA supercoiling of wild-type *Salmonella* and of an engineered strain deleted for the *ydeJ* open reading frame.

**FIG 5:**
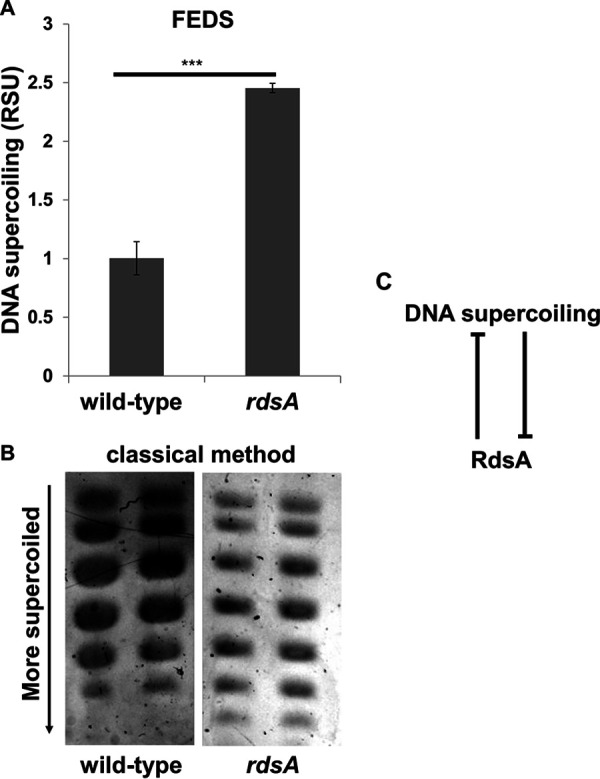
RdsA inhibits DNA supercoiling. (A) DNA supercoiling of wild-type (14028s) and *rdsA* (AAD219) *Salmonella* was measured in 96-well plates using FEDS. (B) DNA supercoiling of the strains described for panel A was measured using the classical agarose/chloroquine gel method. (C) The higher level of DNA supercoiling of the mutant observed with both methods indicates that RdsA is involved in a double-negative-feedback loop with DNA supercoiling. ***, *P* < 0.001 (Student's *t* test).

DNA was more supercoiled in the *ydeJ* mutant than in wild-type *Salmonella* (Fig. 5A and B). This result indicates that the *ydeJ* gene and DNA supercoiling form a double-negative-feedback loop (Fig. 5C). For this reason, we renamed YdeJ “RdsA” (regulator of DNA supercoiling A). *rdsA* encodes a product of unknown biochemical activity (29), and, to our knowledge, no phenotype has been reported for a *rdsA* mutant. Thus, RdsA is a novel regulator of DNA supercoiling.

### FEDS recapitulates known DNA supercoiling behaviors.

We examined the ability of FEDS to report supercoiling phenotypes as first discovered using the agarose/chloroquine gel method. To test the robustness of FEDS, we performed experiments with E. coli, which differs from *Salmonella* in both the DNA supercoiling set point (9) and the absence/presence of DNA binding proteins that compete with nucleoid-associated proteins for binding to the bacterial chromosome (30).

**FIG 6:**
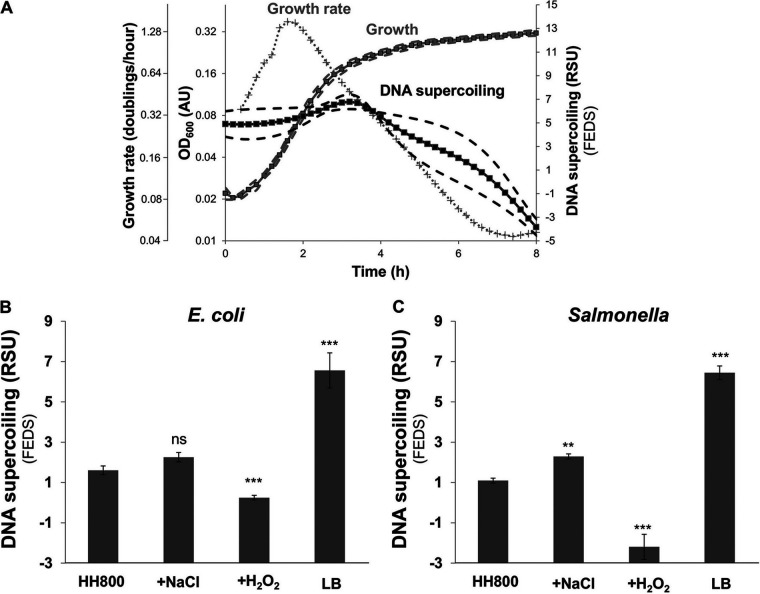
FEDS recapitulates known DNA supercoiling behaviors. (A) DNA supercoiling of wild-type E. coli strain MG1655/pSupR grown in LB in 96-well plates. DNA supercoiling was measured every 12 min using the green/red fluorescence ratio. Data are represented as means (solid lines) ± standard deviations (SD) (dashed lines) of results from 3 replicates. Data representing the average growth rate (dotted gray line) and OD_600_ values (“Growth”; indicated in arbitrary units [AU]) are also plotted. Raw green and red fluorescence data are presented in Fig. S3. (B) DNA supercoiling of E. coli MG1655/pSupR grown in HH800 minimal medium, with NaCl or H_2_O_2_ (where indicated), or in LB broth in 96-well plates as indicated. Conditions were compared when the OD_600_ reached 30% of the maximum OD_600_. Data are represented as means (solid bars) ± SD (error bars) of results from 6 replicates. ns, not significant; ***, *P* < 0.001 (Tukey’s HSD). (C) DNA supercoiling of wild-type *Salmonella* (14028s)/pSupR grown in HH800 minimal medium, with NaCl or H_2_O_2_ (where indicated), or in LB broth in 96-well plates as indicated. Conditions were compared when the OD_600_ reached 30% of the maximum OD_600_. Data are represented as means (solid bars) ± SD (error bars) of results from 6 replicates. **, *P* < 0.01; ***, *P* < 0.001 (Tukey’s HSD).

First, wild-type E. coli K-12 strain MG1655 harboring plasmid pSupR exhibited an increase in the ratio of green to red fluorescence over time as the cells entered stationary growth, reflecting stable negative DNA supercoiling in the exponential phase and then a decrease in negative DNA supercoiling during the early stationary-growth phase (Fig. 6A). This experiment recapitulated the growth-dependent changes in DNA supercoiling at high resolution (Fig. 6A), showing a maximum during the exponential phase and a decrease as E. coli entered the stationary phase (31, 32).

10.1128/mBio.01053-20.4 FIG S3 Comparison of the green fluorescence to red fluorescence ratios in wild-type *S.* Typhimurium (14028s). Download FIG S3, PDF file, 0.1 MB. Copyright © 2020 Duprey and Groisman. 2020 Duprey and Groisman. This content is distributed under the terms of the Creative Commons Attribution 4.0 International license.

Second, the fluorescence ratio of E. coli MG1655/pSupR increased under conditions of exposure to H_2_O_2_, a form of stress that causes DNA relaxation (33), but decreased in response to high osmolarity, a form of stress that causes DNA compaction (34) (Fig. 6B). In addition, the fluorescence ratio was lower during growth in complex media than in defined media (Fig. 6B), reflecting that growth in the former media results in higher DNA negative supercoiling than growth in the latter (31).

And third, experiments carried out with wild-type *S.* Typhimurium strain 14028s harboring pSupR revealed that, as reported previously in E. coli (31, 33, 34), oxidative stress relaxes DNA, whereas osmotic stress and growth in complex media result in compacted DNA (Fig. 6C). These results indicate that E. coli and *Salmonella* respond to specific stimuli by altering DNA supercoiling in similar fashions.

Cumulatively, the results of the experiments described in this section validated the use of pSupR as a DNA supercoiling reporter that functions in two bacterial species that differ in hundreds of genes, including those governing DNA supercoiling (9).

### Single-cell analysis reveals heterogeneous DNA supercoiling behavior.

Certain biological phenomena are revealed only by investigating the behavior of single cells and thus are often missed during measurements of cell populations (35, 36). Although there was no *a priori* reason to suspect that DNA supercoiling would exhibit any particular single-cell behavior, we took advantage of the fact that pSupR specifies two fluorescent proteins to use flow cytometry for high-throughput single-cell measurement of DNA supercoiling.

**FIG 7:**
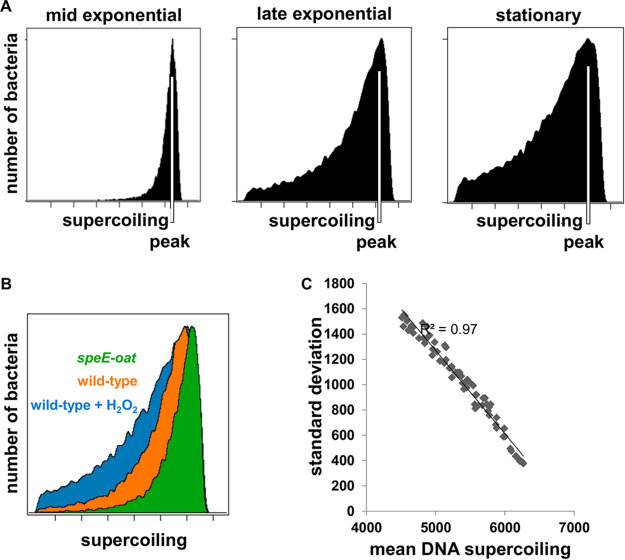
Growth-phase-regulated changes in subpopulations exhibiting different DNA supercoiling behaviors. (A) Density profiles of the wild-type *S.* Typhimurium (14028s)/pSupR strain in LB at different time points. DNA supercoiling was evaluated using the green/red fluorescence ratio produced by the organisms harboring plasmid pSupR. (B) Overlay of density profiles in late exponential phase in HH800 (wild-type strain and isogenic *speE oat* mutant strain AAD58) or HH plus H_2_O_2_ (wild-type strain only). (C) Correlation between population mean and standard deviation across all time points and conditions (14028s/pSupR in LB, HH800, HH, or HH plus H_2_O_2_; isogenic *speE oat* mutant in HH800). Flow cytometry profiles of the populations were recorded every 90 min for 7.5 h.

We determined that the distribution of negative DNA supercoiling is represented by a narrow peak during the early exponential phase of *Salmonella* growth in LB broth (Fig. 7A). As cells enter stationary phase, the peak widens toward relaxed DNA, but, surprisingly, does not change its mode (Fig. 7A). The mean level of DNA supercoiling decreases during growth, while the standard deviation increases. In other words, populations with more relaxed DNA become more variable. This unexpected result suggests that population-level DNA relaxation results from the formation of a relaxed DNA subpopulation coexisting with the highly DNA-supercoiled population (as opposed to originating from a uniform shift of the whole population toward more relaxed DNA).

The phenomenon described above was also observed in comparisons of different conditions rather than of time points. For example, the DNA relaxation caused by oxidative stress widened the peak of fluorescence (Fig. 7B). By contrast, the high level of negative DNA supercoiling of a *speE oat* double mutant resulted in a narrower fluorescence peak than was seen with the wild-type strain (Fig. 7B). More broadly, the means and standard deviations of DNA supercoiling at the single-cell level are highly anticorrelated across time points and conditions (*R*^2^ = 0.97) (Fig. 7C).

## DISCUSSION

We developed FEDS, a method that reports DNA supercoiling *in vivo*. FEDS relies on a plasmid that harbors a promoter responding exclusively to DNA supercoiling controlling transcription of the gene for a green fluorescent protein and a constitutive promoter that drives expression of the gene for a red fluorescent protein. We demonstrated the utility of FEDS to recapitulate known DNA supercoiling behaviors in two enteric bacterial species that differ in their basal DNA supercoiling set points (9), to discover new supercoiling-regulating proteins and promoters, and to unveil single-cell DNA supercoiling heterogeneity.

### FEDS enables high-throughput exploration of DNA supercoiling and opens new possibilities.

We used FEDS successfully to examine supercoiling behaviors in bacteria grown in microtiter plates and a plate reader. Thus, FEDS allows entirely automatic high-throughput and high-temporal-resolution measurement of DNA supercoiling. It is now feasible to carry out genetic or chemical screenings based on DNA supercoiling that were not previously possible. For instance, using 96-well plates, one can reasonably screen 3,000 mutants/compounds every 48 h, which represents a >100-fold increase in throughput compared to the current method (i.e., the use of agarose/chloroquine gels) (12). Such screenings may enable the identification of novel targets for the development of drugs that alter DNA supercoiling and counter the growing bacterial resistance to existing drugs (37).

In addition to its demonstrated utility in flow cytometry (Fig. 7), FEDS is compatible with fluorescence microscopy, which enables direct measurement of bacterial DNA supercoiling during infection. For example, *Salmonella* changes its DNA supercoiling inside macrophages (38). FEDS can facilitate examination of dynamic variations in DNA supercoiling at the single-cell level by direct imaging of live bacteria inside macrophages. Finally, the system is modular and can be adapted to fit a variety of situations in which one wants to measure DNA supercoiling (see Text S1 in the supplemental material).

10.1128/mBio.01053-20.1 TEXT S1 Expanding FEDS to more-distant bacterial species. Download Text S1, DOCX file, 0.1 MB. Copyright © 2020 Duprey and Groisman. 2020 Duprey and Groisman. This content is distributed under the terms of the Creative Commons Attribution 4.0 International license.

### A promoter specifically repressed by DNA supercoiling drives transcription of a negative regulator of DNA supercoiling.

We determined that the activity of the *rdsA* promoter decreased as negative DNA supercoiling increased both *in vivo* and *in vitro* (Fig. 3 and 4; see also Fig. S4 in the supplemental material). Thus, we used the *rdsA* promoter as a reporter of cellular DNA supercoiling. Other supercoiling-responsive promoters had been described previously (39), including those driving transcription of the *topA* and *gyrB* genes, which specify topoisomerase I and one of the two subunits of DNA gyrase (12, 40), respectively. Even though the activity of the *topA* and *gyrB* promoters is regulated by DNA supercoiling, these two promoters are controlled by other factors (41, 42) (see Data Set S1 in the supplemental material), making them unsuitable for use as supercoiling sensors (Fig. S4). What makes the *rdsA* promoter unique is its exclusive regulation by conditions that alter DNA supercoiling.

10.1128/mBio.01053-20.5 FIG S4 Expression of the *topA*, *gyrB*, *rdsA*, and *hupB* genes as a function of conditions that alter DNA supercoiling. DNA supercoiling was measured using the classical agarose/chloroquine gel method. Gene expression was evaluated by RNA-seq. Data for all *Salmonella* genes are presented in Data Set S2. Download FIG S4, PDF file, 0.2 MB. Copyright © 2020 Duprey and Groisman. 2020 Duprey and Groisman. This content is distributed under the terms of the Creative Commons Attribution 4.0 International license.

10.1128/mBio.01053-20.9 DATA SET S1 Expression of all genes in the 14028s genome as a function of DNA supercoiling. Download Data Set S1, XLSX file, 0.8 MB. Copyright © 2020 Duprey and Groisman. 2020 Duprey and Groisman. This content is distributed under the terms of the Creative Commons Attribution 4.0 International license.

10.1128/mBio.01053-20.10 DATA SET S2 Regression analysis summary of the 11-condition RNA-seq experiment. Download Data Set S2, XLSX file, 0.9 MB. Copyright © 2020 Duprey and Groisman. 2020 Duprey and Groisman. This content is distributed under the terms of the Creative Commons Attribution 4.0 International license.

A search for supercoiling-responsive promoters in E. coli identified 306 supercoiling-regulated genes whose expression was altered in the presence of DNA gyrase inhibitors (39). Unfortunately, it is presently unknown whether the corresponding promoters are exclusively regulated by DNA supercoiling or would behave in a similar manner when cloned into a reporter plasmid.

By contrast, because the supercoiling sensor reported here is plasmid based, it was important to identify promoters whose response to DNA supercoiling was the same whether in the chromosome or the reporter plasmid. To identify such promoters, we first used RNA-seq to isolate candidate promoters with supercoiling sensitivity in the chromosome (Fig. 2) and then moved them to a plasmid and verified that they still responded to DNA supercoiling in the same way (Fig. 3). By analyzing DNA supercoiling and RNA abundance genome wide in the same bacterial cultures, we identified genes exclusively regulated by DNA supercoiling (Data Set S2). However, only one of the eight corresponding promoters—that of the *rdsA* gene—retained the regulation by DNA supercoiling once cloned into the reporter plasmid. This finding supports the importance of genomic context in the transcriptional response of genes to DNA supercoiling that had been previously suggested by psoralen cross-linking results (14).

The multicopy plasmid pSupR reports on global, average negative DNA supercoiling. However, the p_ffh_-*tdtomato*-*gfp*-p*_rdsA_* module present in pSupR can be inserted into the chromosome to investigate how genome location impacts DNA supercoiling. Such investigation may reveal DNA supercoiling heterogeneity along the chromosome currently hypothesized based on the transcriptional response to gyrase inhibitors (11).

### Conclusions.

FEDS allows rapid and easy measurement of DNA supercoiling using commonly available equipment and software. The principles governing the construction of pSupR and development of FEDS can be applied to other species, including prokaryotic and eukaryotic organisms in which fluorescent reporters are available. We expect FEDS to pave the way toward understanding the pathways that control DNA supercoiling, its effects on transcription and recombination, and how to disrupt DNA supercoiling in a predictable fashion.

## MATERIALS AND METHODS

All materials and their references are summarized in Table S1A in the supplemental material.

### Bacteria and growth conditions.

S. enterica serovar Typhimurium 14028s and E. coli K-12 MG1655 and isogenic derivatives were used during this work. Strains were grown in HH minimal medium (which is based on N-minimal medium [43]) at 37°C with aeration (shaking at 250 rpm) except otherwise indicated. HH is made of KCl 5 mM, (NH_4_)_2_SO_4_ 7.5 mM, K_2_SO_4_ 0.5 mM, KH_2_PO_4_ 1 mM, Tris 50 mM, bis-Tris 50 mM, MgCl_2_ 10 mM, Casamino Acids 0.1%, glycerol 0.27% (pH 7.7). HH800 is identical to HH but has 800 μM MgCl_2_ instead of 10 mM MgCl_2_. Antibiotics were used at the following concentrations: ampicillin (Amp), 50 μg/ml; chloramphenicol, 25 μg/ml.

E. coli MG1655 was further supplemented with 1 μg/ml biotin and 1 μg/ml thiamine. All constructed plasmids were amplified in E. coli DH5α and grown aerobically in LB at 37°C. A method based on the use of TSS (polyethylene glycol [PEG] 3350 10%, MgCl_2_ 10 mM, MgSO_4_ 10 mM, dimethyl sulfoxide [DMSO] 5%, with LB as the solvent and adjusted to pH 6.3) was used to transform E. coli (44), and electroporation was used to transform *Salmonella* as described below.

### Transformation of E. coli.

Cells (1 ml) were grown in LB to an optical density at 600 nm (OD_600_) of 0.4. The cells were then centrifuged and resuspended in 100 μl cold TSS. DNA or ligation reaction mixture was then added (about 10 ng supercoiled DNA or 50 ng ligation reaction mixture). Cells were incubated on ice for 30 min, heat shocked at 42°C for 50 s, and left on ice for 2 min. LB (900 μl) was added, and the cells were incubated at 37°C for 1 h before plating on selective medium was performed.

### Electroporation of *S.* Typhimurium.

LB medium was used throughout the experiments. Per electroporation, a 5-ml cell volume was inoculated with 50 μl of saturated preculture, and the cells were then grown at 37°C for 3.5 h. The cells were then washed three times in cold water and resuspended into 50 μl cold water, and 100 ng of plasmid DNA was added. Electroporation was performed using a Gene Pulser II electroporator (Bio-Rad) (25 μF and 1.7 kV). A 1-ml volume of LB was added, and cells were incubated at 37°C for 1 h before plating on selective medium was performed.

### Strain construction.

Mutations were created by the λred recombination method (45). pSIM6 was used to supply λred. The electroporation protocol described above was modified as follows. The strains were grown at 30°C for 3.5 h and then heat shocked at 42°C for 20 min, 500 ng of linear recombinant DNA was used, and SOC (Bacto tryptone 2%, yeast extract 0.5%, NaCl 10 mM, KCl 2.5 mM, MgSO4 10 mM, glucose 20 mM, pH 7) was used at the recovery stage.

After PCR verification of the strains, mutations were transduced into wild-type *S.* Typhimurium strain 14028s using phage P22-mediated transduction (46). Strain AAD46 was built by λred recombination using pKD3 as the template and primer pair 16651/16652. Strain AAD58 was built by P22 transduction using a lysate prepared in strain AAD46 to infect strain JY979. Strain AAD219 was built by λred recombination using pKD3 as the template and primer pair 17365/17366.

### Plasmid construction.

Plasmids were constructed using restriction-ligation approaches. Restriction enzymes, T4 DNA ligase, and Klenow fragments were used according to the manufacturer’s instructions. Detailed step-by-step construction procedures are presented in the following paragraph.

Primer annealing was performed as follows. A 200-pmol volume of each primer was mixed into Tris-EDTA (pH 8)–NaCl 50 mM. The mixture was then heated at 95°C for 5 min and cooled to room temperature slowly (40 min). The annealed primers were then diluted 1/100 (vol/vol) in water. A 0.16-ng volume of annealed primer was used per 30-μg volume of vector in subsequent ligation reactions.

Regulatory regions are defined as the 250-bp fragments surrounding the transcription start site identified in the RNA-seq analysis, corresponding to 200 bp upstream of the transcription start site and 50 bp downstream of it, unless otherwise indicated.

To construct pFPv25-H, the *ydeJ* regulatory region was amplified from wild-type *S.* Typhimurium strain 14028s genomic DNA using primers 16920/16921, purified, digested using EcoRI plus SpeI, and then ligated into pFPv25 (EcoRI plus XbaI) and transformed into DH5α by the use of the TSS method.

*tdtomato* was subjected to codon optimization for expression in *S.* Typhimurium strain LT2 using Jcat and then synthesized by Thermo Fisher Scientific and supplied cloned into plasmid pMK. *tdtomato* was then subcloned (EcoRI plus PstI) into pJV and transformed into DH5α by the use of the TSS method, yielding pJT.

To construct pJTL, the *ffh* promoter from 14028s (−50 to +5 relative to the transcription start site) was obtained by annealing primers 16981/16982. The annealed primers were then cloned into pJT (EcoRI plus XbaI) and transformed into DH5α by the use of the TSS method.

Finally, pJTL was cut with EcoRI plus PstI, filled in with Klenow fragments, and subjected to blunt cloning into pFPv25-H at the EcoRV site, yielding pSupR. The convergent orientations of *gfp* and *tdtomato* were confirmed by restriction mapping.

Plasmids pJTI, pJTJ, and pJTK were constructed as described for pJTL above. The primer pairs used were 16975/16976, 16977/16978, and 16979/16980, respectively. The sequence for *imp* was taken from the 14028s genome, and the sequences for J23100 and J23119 were taken from the registry of standard parts (http://parts.igem.org/Main_Page).

pFPv25-A through pFPv25-G were constructed as described for pFPv25-H above, using primers 16906 through 16919 as described in Table S1.

pFPv25-I and pFPv25-L were constructed by annealing of primers 16975/16976 and 16981/16982, respectively, into pFPv25 (digested with EcoRI plus XbaI).

pFTL was constructed as described for pSupR, except that pFPv25 was used (instead of pFPv25-H).

pFITI, pFITJ, pFITK, and pFITL were constructed as described for pSupR, using pFPv25-I as a vector and blunted pJTI, pJTJ, pJTK, or pJTL as an insertion. pFPv25-L was used as the vector and blunted pJTL as the insertion for pFLTL.

### Considerations concerning *in vivo* DNA supercoiling.

The research presented here concerns only negative DNA supercoiling. Therefore, relaxed DNA (i.e., supercoiling corresponding to values closer to 0) is referred to as “low supercoiling” and as having low values (quantified as either linking number [Lk] or relative supercoiling unit [RSU] values). In contrast, highly supercoiled DNA (i.e., strongly negative DNA supercoiling) is referred to as “high supercoiling” and has high values (in either Lk or RSU units).

### Measurement of DNA supercoiling on agarose/chloroquine gels.

Strains bearing plasmid pJV were precultured overnight in HH plus Amp, washed once in water, and then diluted into appropriate medium (Table 1; see also Table S1B) to a starting OD_600_ of 0.05. Cells were grown until they reached 30% of the saturation OD (OD_600_ =  0.8 ± 0.1 for the wild-type strain), and then plasmids were immediately extracted using a Qiagen Plasmid minikit. An 800-ng volume of purified plasmid for each sample was then loaded on a Tris-borate-EDTA–0.8% agarose–2.5 μg/ml chloroquine gel. Gels were migrated overnight at 1.3 V/cm, washed in water for at least 4 h, and then stained using EZ-vision (VWR) and imaged with an ImageQuant LAS 400 imager (GE Healthcare).

The intensity of each band was quantified with ImageJ. The linking number (Lk) value for the top band was arbitrarily set to 0, and then the value for the band immediately below was Lk = 1, that for the next one Lk = 2, and so on. The intensity-weighted average Lk value was calculated for each lane. The measured DNA supercoiling was normalized across experiments to the supercoiling exhibited by wild-type *S.* Typhimurium strain 14028s following growth in HH, quantified as 0 relative supercoiling units (RSU), and the supercoiling in the WT in HH800, defined as 1 RSU.

The method by which RSUs are calculated from gels is shown in Fig. S5. All raw images of gels used in this work are available at https://doi.org/10.17632/h6g4dkw6sw.1. pSupR could not be used with this method due to its large size and low extraction yields.

10.1128/mBio.01053-20.6 FIG S5 Detailed graphical example of calculation of RSUs from a gel image. (Step 1 [optional]) If the background has strong nonlinear variations, use the “subtract background” function in ImageJ. (Step 2) In ImageJ, draw a measurement area the size of the biggest band. Then, without changing it, measure the mean intensity of each band (“Measure” function). Depending on the image capture software and file format, white may have value 0 (like this example) or 255. This does not impact the rest of the analysis. (Step 3) Calculate the background for subtraction by interpolating the top and bottom blanks linearly. (Step 4) Subtract background from raw intensities. (Step 5) Assign Lk (linking number) values to each band (by arbitrary convention, the top one should be 0, but it has no impact on results). Then, calculate the average Lk value weighted by the subtracted intensities. (Step 6 [for *in vivo* experiments only]) Take the calculated Lk value from wild-type *Salmonella* grown in HH or HH800 media at 37°C to an OD_600_ of 0.8 loaded on the same gel. Linearly transform the Lk values into RSU values such that HH has a value of 0 RSU and HH800 has a value of 1 RSU. The “raw” gel image presented here is an edited composite image used for demonstration purposes only. Actual gels may show up to 6 bands per kilobase plasmid size. Download FIG S5, PDF file, 1.1 MB. Copyright © 2020 Duprey and Groisman. 2020 Duprey and Groisman. This content is distributed under the terms of the Creative Commons Attribution 4.0 International license.

### RNA-seq and scoring of exclusively supercoiling-dependent promoters.

Total RNA was extracted from the same cultures as those used for DNA supercoiling, at the same time, using a Qiagen RNeasy minikit. In addition to the included DNase treatment in the kit, DNA was further eliminated by treatment with Turbo DNase (Ambion). RNA was finally repurified using a Qiagen RNeasy minikit. RNA amounts were quantified by UV absorbance and verified by loading on a Tris-borate-EDTA–1% agarose gel. Because the focus of this experiment was on correlating gene expression to negative DNA supercoiling across many conditions, it was more desirable to have more conditions than more replicates of the same condition; therefore, *n* = 1 for all conditions.

The following operations were performed at the Yale Center for Genome Analysis. rRNA was depleted using a RiboZero kit (Illumina). cDNA synthesis was performed by adding A bases to the 3′ end of fragments, followed by oligo(dT) priming. The 11 samples were barcoded and multiplexed into a single flow cell. DNA sequencing was performed by the use of a HiSeq 4000 sequencer (Illumina) (75 × 2 paired ends, unstranded). Sequencing yielded 25 to 30 million total reads.

After sequencing, reads were mapped to the wild-type *S.* Typhimurium strain 14028s genome (GenBank accession no. CP001363.1) using bowtie (20 to 25 million uniquely mapped paired reads per sample) and differential expression analysis was performed using cuffdiff from the cufflinks package. Default parameters were used in both cases.

Gene expression calculated by cufflinks was used as a base for linear and logistic regressions. Then, for each gene, linear regression (lm function in core R) or logistic regression (G.4 function in drm package for R) was performed.

The amplitude was defined as the difference between the maximum expression level and the minimum expression level after fitting.

To obtain the fitting score, the root mean square sum of the residuals was computed and divided by the amplitude. The fitting score was inversely proportional to this ratio, using arbitrary constants that produced a score between 0 and 3 (a score of 3 represents a root mean square sum of residuals equal to 0 and, as a result, was never achieved). To avoid aberrant sigmoidal regressions, the fitting score was penalized and set to −0.1 if no experimental points matched the upper asymptote. This eliminated genes that are not expressed except under one set of conditions; such genes were unsuitable as reporters but tended to score highly on the logistic regression.

The mean expression score was 1 for all genes above a value of 2,000 FPKM (fragments per kilobase per million) and then decreased linearly to 0 as gene expression decreased to 0 FPKM.

The amplitude score was the amplitude divided by the maximum expression level after fitting.

The overall score was the sum of the fitting score, mean expression score, and amplitude score. Therefore, the fitting score had triple the weight of each of the other scores, because a good fit was absolutely essential for the system to work. In contrast, the effects of a moderately bad mean level of expression or amplitude could be circumvented by other approaches (such as the use of a stronger ribosome binding site or brighter fluorescent proteins for determination of the mean expression level).

The true-positive rate was estimated by repeating the same analysis using a random subset of the 11 conditions used for the RNA-seq. For each *n* (*n* > 2) quantity of conditions, data from *n* conditions were selected randomly and the number of genes passing the cutoff score of 2.9 was computed. For each *n*, the average number of genes passing cutoff *y* was then plotted and fitted to a 4-parameter power law as follows:

$$y\left(n\right)=N+a{\left(c-n\right)}^{b}$$

With *b* values of <0, this function converges to *N*, which represents the quantity of true positives.

### FEDS.

Strains bearing plasmid pSupR were cultured overnight in HH defined media containing ampicillin, washed once in water, and then diluted into appropriate medium (Fig. 3, 5, and 7) to a starting OD_600_ of 0.05 into either flasks (Fig. 3 and 7) or 96-well plates (Fig. 5).

Flask cultures were grown as described above. Samples were taken regularly (every 1 to 2 h), and OD_600_ and fluorescence were measured as described below.

Growth of cultures in a 96-well plate was performed at 37°C with discontinuous agitation. The positioning of the different samples in the plate was randomized. Each plate had two different blanks: one with HH medium and one with LB medium. The plate was agitated in a linear trajectory (20 s, 3 mm, 50 rpm) every 12 min. OD_600_ and fluorescence were measured at the end of each agitation cycle.

All OD_600_ measurements were performed in a BioPhotometer (Eppendorf) for flasks or in an Infinite M1000 reader (Tecan) for plates. Fluorescence measurements were performed using an Infinite M1000 reader (Tecan). For Gfp, the excitation wavelength was 485.0 ± 2.5 nm and the emission wavelength was 530.0 ± 2.5 nm. For tdTomato, the excitation wavelength was 550.0 ± 2.5 nm and the emission wavelength was 580.0 ± 2.5 nm.

Raw OD_600_ and fluorescence data were processed as follows. First, the measurements for the blanks were subtracted. Fluorescence for each color was further normalized to the fluorescence of LB for the corresponding color. Then, the fluorescence data were smoothed by linear regression to a degree 12 polynomial. Ratios of green fluorescence over red fluorescence were calculated. Wild-type *S.* Typhimurium strain 14028s grown in HH or HH800 was present in all experiments, allowing the conversion of these ratios to values representing DNA supercoiling expressed in RSU, where supercoiling in HH medium at 30% of the maximum OD_600_ is 0 RSU and supercoiling in HH800 medium at 30% of the maximum OD_600_ is 1 RSU.

This approach using OD_600_ values expressed as a percentage of the maximum (rather than using time points or defined OD_600_ values) allows relevant comparisons of conditions and of strains with different growth rates. Notably, the strains bearing pJV and those bearing pSupR had different growth profiles (see Fig. S6 in the supplemental material).

10.1128/mBio.01053-20.7 FIG S6 Growth of wild-type *S.* Typhimurium strain 14028s bearing plasmid pSupR or plasmid pJV. OD_600_ of wild-type *S.* Typhimurium (14028s) harboring plasmid pSupR or plasmid pJV grown in HH800 medium in 96-well plates. Data are represented as means (solid lines) ± SD (dashed lines) of results from 3 replicates. Download FIG S6, PDF file, 0.1 MB. Copyright © 2020 Duprey and Groisman. 2020 Duprey and Groisman. This content is distributed under the terms of the Creative Commons Attribution 4.0 International license.

### Correlation clustering test.

Wild-type *S.* Typhimurium strain 14028s carrying plasmid pFITI, pFITJ, pFITK, or pFITL was grown in flasks as described above by the use of the FEDS method. The conditions used were as follows: HH plus novobiocin 25 μg/ml, HH, HH plus NaCl 300 mM, HH plus FeSO_4_ 100 μM, and LB. Six time points were measured.

Fluorescent protein stability and dilution at division were corrected using the following formula for stable fluorescent proteins (47):

$$r\left(t\right)=\frac{\mathrm{dq}(t)}{\mathrm{dt}}+\text{μ}\left(t\right)q(t)$$

where *r*(*t*) represents the concentration of transcript (corrected for protein stability and dilution) in arbitrary units, *q*(*t*) is the fluorescence/OD ratio, and μ(*t*) is growth rate.

Linear regressions were then performed, and correlation coefficients (*R*^2^) were calculated.

### *In vitro* transcription.

pSupR was extracted from DH5α using a Qiagen maxi kit. Then, the plasmid was treated for 2 h with either DNA topoisomerase I or XmnI in CutSmart buffer according to the manufacturer’s instructions, yielding the relaxed plasmid or the linearized plasmid, respectively. Enzymes were then subjected to heat inactivation. The supercoiled plasmid was obtained by diluting the raw plasmid extract into CutSmart buffer to reach the same concentration as that of the treated plasmid.

The transcription reaction was performed using E. coli RNA polymerase (RNAP) from NEB. A 150-ng volume of plasmid and 0.5 U RNAP were used in 10-μl reaction mixtures according to the manufacturer’s instructions, and the mixtures were incubated for 1 h at 37°C. The reaction was stopped by heat inactivation.

Transcript detection was performed by the use of qRT-PCR with tailed primers (48). DNA was first eliminated by adding 1 μl eZDNase buffer and 0.4 μl eZDNase to the previously described reaction mixtures and incubating at 37°C for 20 min. DNase was then inactivated by treatment with 10 mM dithiothreitol (DTT) at 55°C for 5 min. Reverse transcription was performed using a SuperScript IV first-strand kit. A 1-μl volume of a mixture of primers 17660 and 17661 (4 μM each) was used as a primer. Quantitative PCR (qPCR) was performed using SYBR green master mix and primer pair 17662/17663 (*gfp*) or primer pair 17664/17665 (*tdtomato*).

### Flow cytometry.

Cells were grown in flasks according to the FEDS method, diluted in water to an OD_600_ of about 0.03, and then injected into a FACSCalibur apparatus (BD). The excitation laser was used at 488/10 nm. FL1 (530/30 nm) was used to record Gfp fluorescence, and FL2 (585/42 nm) was used to record tdTomato fluorescence.

Wild-type 14028s (i.e., not carrying pSupR) was used as a nonfluorescent control. 14028s/pFPv25-H was used as a pure green and DH5α/pFTL was used as a pure red for compensation purposes.

For each event, green/red ratios were calculated. Ratios were converted to DNA supercoiling using the following empirical formula: supercoiling = 150 × (45 − ratio). A custom R script was used to append these data to the FL3 channel of the fcs files. The modified fcs files were then analyzed using Cytobank. The data can be publicly accessed at https://community.cytobank.org/cytobank/experiments/84784/illustrations/155187.

### General statistical procedures.

Data are represented as means ± standard deviations; *n* represents the number of independent bacterial cultures.

Tukey’s honestly significant difference (HSD) test or Student's *t* test was used for statistical analysis as indicated in the figure legends, where *P* values of <0.05 were considered significant.

### Data availability.

The raw data associated with this paper are available in Mendeley (https://data.mendeley.com/datasets/h6g4dkw6sw/2).
