# Correction for Duprey and Groisman, “FEDS: a Novel Fluorescence-Based High-Throughput Method for Measuring DNA Supercoiling *In Vivo*”

**DOI:** 10.1128/mbio.02684-23

**Published:** 2023-12-07

**Authors:** Alexandre Duprey, Eduardo A. Groisman

## AUTHOR CORRECTION

Volume 11, no. 4, e01053-20, 2020, https://doi.org/10.1128/mBio.01053-20: In our paper, the promoter driving transcription of the *tdtomato* gene in pSupR is said to correspond to the *ffh* gene. However, this is not correct. This promoter corresponds to the *yffH* (also known as *nudK*) gene. Importantly, the mRNA abundance of the *yffH* gene is as unresponsive as that of the *ffh* gene to the 11 investigated conditions that change DNA supercoiling. Therefore, the conclusions of the paper and the utility of pSupR as a reporter of DNA supercoiling *in vivo* remain unchanged. A complete description of pSupR is available from GenBank (accession number OR650783). We thank Dr. Nicholas Pokorkynski (Yale School of Medicine) for bringing the error in our paper to our attention. We apologize to the scientific community for any inconvenience the error may have caused.

