# Supplementary material for: FEDS: a Novel Fluorescence-Based High-Throughput Method for Measuring DNA Supercoiling In Vivo
Source: mBio. 2020 Jul 28;11(4):e01053-20. doi: 10.1128/mBio.01053-20 (PMC7387798; doi:10.1128/mBio.01053-20)
Supplement: FIG S1 [file mBio.01053-20-sf001.pdf]

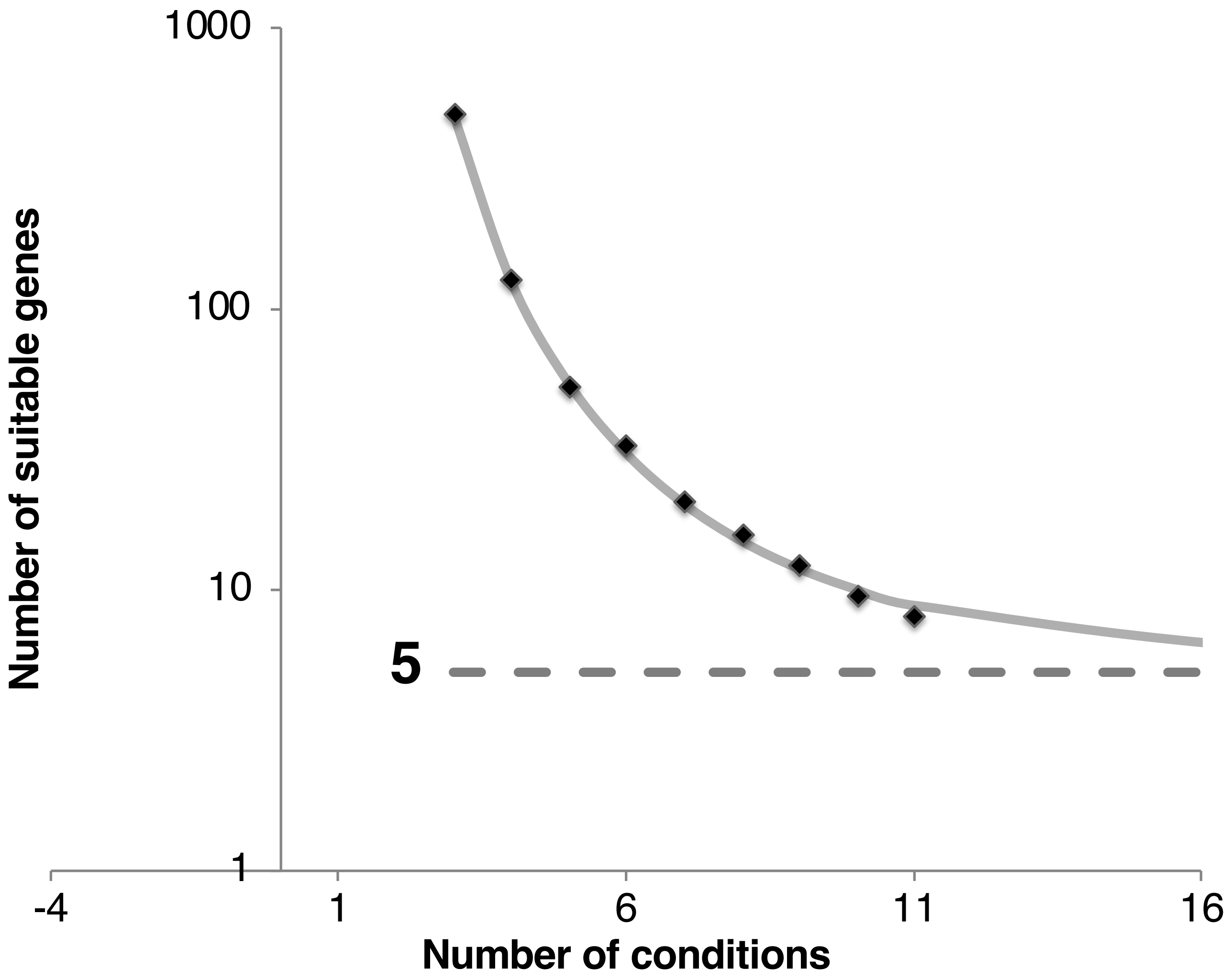

Figure S1: Estimation of the true positive rate given by the number of conditions used in the RNA-seq experiment.

Data from the n conditions (*x* axis) out of the 11-condition RNA-seq experiment were randomly shuffled and the number of genes passing the cutoff score of 2.9 was computed. The average number of genes passing the cutoff was then fitted to a power law that asymptotically converges to the number of expected true positives (5 in this case).
