# Supplementary material for: FEDS: a Novel Fluorescence-Based High-Throughput Method for Measuring DNA Supercoiling In Vivo
Source: mBio. 2020 Jul 28;11(4):e01053-20. doi: 10.1128/mBio.01053-20 (PMC7387798; doi:10.1128/mBio.01053-20)
Supplement: TABLE S1 [file mBio.01053-20-st001.docx]

Table S1A: Summary of materials used in this study

| DESCRIPTION | SOURCE | IDENTIFIER |
| --- | --- | --- |
| Bacterial and Virus Strains | | |
| Phage for *S.* Typhimurium | (1) | P22 |
| *E. coli* |  |  |
| Cloning strain |  | DH5α |
| Wild-type |  | MG1655 |
| *S. Typhimurium* |  |  |
| Wild-type | (2) | 14028s |
| *hupB::Tn10* | A. Sevastyanova, personal communication | AS101 |
| *fis::cat* | E. Fass, personal communication | EF259 |
| *ΔmgtC* | (3) | EL4 |
| *speE::cat Δoat* | This work | AAD58 |
| *pmrA::cat* | (4) | EG7139 |
| *Δoat* | J. Yeom, personal communication | JY979 |
| *speE::cat* | This work | AAD46 |
| *rdsA::cat (= ydeJ::cat)* | This work | AAD219 |
| Chemicals, Peptides, and Recombinant Proteins | | |
| DNA polymerase, Klenow fragment | NEB  (MA, USA) | M0210 |
| T4 DNA ligase | NEB  (MA, USA) | M0202 |
| EcoRI-HF | NEB  (MA, USA) | R3101 |
| EcoRV-HF | NEB  (MA, USA) | R3195 |
| PstI-HF | NEB  (MA, USA) | R3140 |
| XbaI | NEB  (MA, USA) | R0145 |
| XmnI | NEB  (MA, USA) | R0914 |
| DNA topoisomerase I | NEB  (MA, USA) | M0301 |
| *E. coli* RNA polymerase, holoenzyme | NEB  (MA, USA) | M0551 |
| Novobiocin | Sigma  (MO, USA) | N1628 |
| Ampicillin | Sigma  (MO, USA) | A9518 |
| Chloramphenicol | Sigma  (MO, USA) | C0378 |
| Critical Commercial Assays | | |
| Plasmid mini | QIAGen  (Germany) | 27106 |
| Plasmid maxi | QIAGen  (Germany) | 12163 |
| RNeasy mini | QIAGen  (Germany) | 74106 |
| eZDNase | Thermo-Fisher  (MA, USA) | 11766051 |
| Superscript IV first strand kit | Thermo-Fisher  (MA, USA) | 18091050 |
| SYBR Green Master Mix | Thermo-Fisher  (MA, USA) | 4385610 |
| Deposited Data | | |
| RNA-seq raw data | GEO | GSE137586 |
| Oligonucleotides | | |
| aattcgcggccgcttctagagTTGACGGCTAGCTCAGTCCTAGGTACAGTGCTAGCta | Keck oligos | 16975 (J23100_FW) |
| ctagtaGCTAGCACTGTACCTAGGACTGAGCTAGCCGTCAActctagaagcggccgcg | Keck oligos | 16976 (J23100_RV) |
| aattcgcggccgcttctagagTTTATGGCTAGCTCAGTCCTAGGTACAATGCTAGCta | Keck oligos | 16977 (J23114_FW) |
| ctagtaGCTAGCATTGTACCTAGGACTGAGCTAGCCATAAActctagaagcggccgcg | Keck oligos | 16978 (J23114_RV) |
| aattcgcggccgcttctagagGAAGGCTACGGTCAATTGTGGGACATCGCCTATAATTTGCATTATCATACCTGTCta | Keck oligos | 16979 (imp_FW) |
| ctagtaGACAGGTATGATAATGCAAATTATAGGCGATGTCCCACAATTGACCGTAGCCTTCctctagaagcggccgcg | Keck oligos | 16980 (imp_RV) |
| aattcgcggccgcttctagagCCTTCAGCAGACAAAACCGACCTCACGGCGTAATATTAATGCTCTTTTTACACCCta | Keck oligos | 16981 (ffh_FW) |
| ctagtaGGGTGTAAAAAGAGCATTAATATTACGCCGTGAGGTCGGTTTTGTCTGCTGAAGGctctagaagcggccgcg | Keck oligos | 16982 (ffh_RV) |
| ATTATGTTGCGCCCTTTTTTTACGGGTGTTAACAAAGGAGGTATCAACCCGTGTAGGCTGGAGCTGCTTC | Keck oligos | 16651 (speE_FW) |
| AGATTATTAAAGCCATGCAGTTTCAGTTTTTTCAATTTCTTATCTTCTCCCATATGAATATCCTCCTTAG | Keck oligos | 16652 (speE_RV) |
| aaagaattcgcggccgcttctagagGGTTTGGTTGACTCAATTTTG | Keck oligos | 16906 (0531_FW) |
| aaactgcagcggccgctactagtaACGACGCAAATGCCATTCTC | Keck oligos | 16907 (0531_RV) |
| aaagaattcgcggccgcttctagagGCTGGCGCATTCGCTTAG | Keck oligos | 16908 (2665_FW) |
| aaactgcagcggccgctactagtaGCAGGAAAAGGGCGATCAC | Keck oligos | 16909 (2665_RV) |
| aaagaattcgcggccgcttctagagAACCTTCCATTTCGAGGAGC | Keck oligos | 16910 (cpxP_FW) |
| aaactgcagcggccgctactagtaAACAGCAGCGGTAACTTTGC | Keck oligos | 16911 (cpxP_RV) |
| aaagaattcgcggccgcttctagagTTTCCACATTTAATATTAATTTATGTTG | Keck oligos | 16912 (gcvT_FW) |
| aaactgcagcggccgctactagtaAAATCTTCGCCACGAAACCTG | Keck oligos | 16913 (gcvT_RV) |
| aaagaattcgcggccgcttctagagATTGCCGATCTGGATATTCATC | Keck oligos | 16914 (hupB_FW) |
| aaactgcagcggccgctactagtaAGTTATATCAGGCCTGCCAC | Keck oligos | 16915 (hupB_RV) |
| aaagaattcgcggccgcttctagagACTCCTTGAAAAGTAAAGTGTTAG | Keck oligos | 16916 (maeB_FW) |
| aaactgcagcggccgctactagtaGTATCGTTCAAAAAGTGACGC | Keck oligos | 16917 (maeB_RV) |
| aaagaattcgcggccgcttctagagTCTTCGTCGTCTTTTGGGC | Keck oligos | 16918 (rbfA_FW) |
| aaactgcagcggccgctactagtaGCGCTGAGGGCGACC | Keck oligos | 16919 (rbfA_RV) |
| aaagaattcgcggccgcttctagagGTCATATGCATCAACGTCTG | Keck oligos | 16920 (ydeJ_FW) |
| aaactgcagcggccgctactagtaCTcatCGTTTATTTTTCCGTTAC | Keck oligos | 16921 (ydeJ_RV) |
| TTGAGAAGAACGAACAGATTTCATTTGTCTCTGTAACGGAAAAATAAACGGTGTAGGCTGGAGCTGCTTC | Keck oligos | 17365 (ydeJ_F) |
| TTTTTTATTCTGACCGGGAGTTATTCTGACCGGTCAGATGAAATTATTGTCATATGAATATCCTCCTTAG | Keck oligos | 17366 (ydeJ_R) |
| CGGTTTGCTAGTTCACGATGGTCTGCTAGTTGAAC | Keck oligos | 17660 (Gfp_RT_R) |
| ACCTCTAAGTAAGTGAGAGAGCCGGTAGAGCC | Keck oligos | 17661 (tdT_RT_F) |
| GTTTGCTAGTTCACGATGGTC | Keck oligos | 17662 (g_qtail_F) |
| TGGCAGACAAACAAAAGAATGG | Keck oligos | 17663 (g_qtail_R) |
| TCTAAGTAAGTGAGAGAGCCG | Keck oligos | 17664 (t_qtail_F) |
| GGACATCACCTCTCACAACG | Keck oligos | 17665 (t_qtail_R) |
| Recombinant DNA | | |
| pMK with a promoterless *tdTomato* | Thermo-Fisher  (MA, USA) | pMK-tdtomato |
| Vector bearing a promoterless *gfp* | (5) | pFPv25 |
| Standard vector for DNA supercoiling measurements | (6) | pJV |
| pFPv25 with the *rdsA* promoter controlling *gfp* and the *ffh* promoter controlling *tdtomato* | This work | pSupR |
| pFPv25 with a promoterless *gfp* and the *ffh* promoter controlling *tdtomato* | This work | pFTL |
| pFPv25 with the STM14_0531 promoter controlling *gfp* | This work | pFPv25-A |
| pFPv25 with the STM14_2665 promoter controlling *gfp* | This work | pFPv25-B |
| pFPv25 with the *cpxP* promoter controlling *gfp* | This work | pFPv25-C |
| pFPv25 with the *gcvT* promoter controlling *gfp* | This work | pFPv25-D |
| pFPv25 with the *hupB* promoter controlling *gfp* | This work | pFPv25-E |
| pFPv25 with the *maeB* promoter controlling *gfp* | This work | pFPv25-F |
| pFPv25 with the *rbfA* promoter controlling *gfp* | This work | pFPv25-G |
| pFPv25 with the *rdsA* promoter controlling *gfp* | This work | pFPv25-H |
| pFPv25 with the J23100 promoter controlling *gfp* | This work | pFPv25-I |
| pFPv25 with the *ffh* promoter controlling *gfp* | This work | pFPv25-L |
| pJV with a promoterless *tdtomato* | This work | pJT |
| pJT with the J23100 promoter controlling *tdtomato* | This work | pJTI |
| pJT with the J23119 promoter controlling *tdtomato* | This work | pJTJ |
| pJT with the *imp* promoter controlling *tdtomato* | This work | pJTK |
| pJT with the *ffh* promoter controlling *tdtomato* | This work | pJTL |
| pFPv25 with the J23100 promoter controlling *gfp* and the J23100 promoter controlling *tdtomato* | This work | pFITI |
| pFPv25 with the J23100 promoter controlling *gfp* and the J23119 promoter controlling *tdtomato* | This work | pFITJ |
| pFPv25 with the J23100 promoter controlling *gfp* and the *imp* promoter controlling *tdtomato* | This work | pFITK |
| pFPv25 with the J23100 promoter controlling *gfp* and the *ffh* promoter controlling *tdtomato* | This work | pFITL |
| pFPv25 with the *ffh* promoter controlling *gfp* and the *ffh* promoter controlling *tdtomato* | This work | pFLTL |
| λred plasmid, heat-inducible | (7) | pSIM6 |
| Template vector for λred recombination, Cm^R^ | (8) | pKD3 |
| Software and Algorithms | | |
| Jcat | (9) | 2008 |
| Cytobank | https://community.cytobank.org/ | 7.2 |
| Bowtie | (10, 11) | 2.2.9 |
| Cufflinks | (12, 13) | 2.2.1 |
| Cuffdiff | (14) | 2.1.1 |
| drm | (15) | 3.0-1 |
| R | (16) | 3.5.2 |
| Other | | |
| 14028s gDNA sequence | GenBank | CP001363.1 |

Table S1B: Description of the 15 conditions used to validate pSupR

| Genotype | Medium | Measured DNA supercoiling (RSU) |
| --- | --- | --- |
| *WT* | HH + 25 μg/mL novobiocin | -2.15 |
| *WT* | HH + 0.2 mM H_2_O_2_ | -1.45 |
| *WT* | HH | 0.00 |
| *hupB* | HH | 0.26 |
| *fis* | HH | 0.54 |
| *mgtC* | HH800 | 0.72 |
| *WT* | HH800 | 1.00 |
| *WT* | HH800 pH 4.6 | 1.23 |
| *WT* | HH800 + 300 mM NaCl | 1.46 |
| *WT* | HH + 100 μM FeSO_4_ | 1.64 |
| *WT* | HH, 10 μM Mg^2+^ | 2.00 |
| *WT* | HH, 10 μM Mg^2+^, pH 4.6 | 2.49 |
| *pmrA* | HH800 | 3.14 |
| *speE-oat* | HH800 | 3.99 |
| *WT* | LB | 5.31 |

Blue text indicates a condition that was present in the 11-condition RNA-seq, magenta text indicates a new condition.

**REFERENCES**

1. Davis RW, Botstein D, Roth JR. 1980. Advanced Bacterial Genetics: A Manual for Genetic Engineering. Cold Spring Harbor Laboratory Pr, Cold Spring Harbor, N.Y.

2. Fields PI, Groisman EA, Heffron F. 1989. A Salmonella locus that controls resistance to microbicidal proteins from phagocytic cells. Science 243:1059–1062.

3. Lee E-J, Pontes MH, Groisman EA. 2013. A Bacterial Virulence Protein Promotes Pathogenicity by Inhibiting the Bacterium’s Own F1Fo ATP Synthase. Cell 154:146–156.

4. Soncini FC, Véscovi EG, Solomon F, Groisman EA. 1996. Molecular basis of the magnesium deprivation response in Salmonella typhimurium: identification of PhoP-regulated genes. J Bacteriol 178:5092–5099.

5. Valdivia RH, Falkow S. 1996. Bacterial genetics by flow cytometry: rapid isolation of Salmonella typhimurium acid-inducible promoters by differential fluorescence induction. Mol Microbiol 22:367–378.

6. Duprey A, Muskhelishvili G, Reverchon S, Nasser W. 2016. Temporal control of Dickeya dadantii main virulence gene expression by growth phase-dependent alteration of regulatory nucleoprotein complexes. Biochim Biophys Acta 1859:1470–1480.

7. Datta S, Costantino N, Court DL. 2006. A set of recombineering plasmids for gram-negative bacteria. Gene 379:109–115.

8. Datsenko KA, Wanner BL. 2000. One-step inactivation of chromosomal genes in Escherichia coli K-12 using PCR products. Proc Natl Acad Sci 97:6640–6645.

9. Grote A, Hiller K, Scheer M, Münch R, Nörtemann B, Hempel DC, Jahn D. 2005. JCat: a novel tool to adapt codon usage of a target gene to its potential expression host. Nucleic Acids Res 33:W526–W531.

10. Langmead B, Salzberg SL. 2012. Fast gapped-read alignment with Bowtie 2. Nat Methods 9:357–359.

11. Langmead B, Trapnell C, Pop M, Salzberg SL. 2009. Ultrafast and memory-efficient alignment of short DNA sequences to the human genome. Genome Biol 10:R25.

12. Trapnell C, Williams BA, Pertea G, Mortazavi A, Kwan G, Van MB, Salzberg SL, Wold BJ, Pachter L. 2010. Transcript assembly and quantification by RNA-Seq reveals unannotated transcripts and isoform switching during cell differentiation. Nat Biotechnol 28:511–515.

13. Trapnell C, Roberts A, Goff L, Pertea G, Kim D, Kelley DR, Pimentel H, Salzberg SL, Rinn JL, Pachter L. 2012. Differential gene and transcript expression analysis of RNA-seq experiments with TopHat and Cufflinks. Nat Protoc 7:562–578.

14. Trapnell C, Hendrickson DG, Sauvageau M, Goff L, Rinn JL, Pachter L. 2013. Differential analysis of gene regulation at transcript resolution with RNA-seq. Nat Biotechnol 31:46–53.

15. Ritz C, Baty F, Streibig JC, Gerhard D. 2015. Dose-Response Analysis Using R. PLOS ONE 10:e0146021.

16. R Core Team. 2018. R: A language and environment for statistical computing. R Found Stat Comput Vienna Austria.
