# Supplementary material for: FEDS: a Novel Fluorescence-Based High-Throughput Method for Measuring DNA Supercoiling In Vivo
Source: mBio. 2020 Jul 28;11(4):e01053-20. doi: 10.1128/mBio.01053-20 (PMC7387798; doi:10.1128/mBio.01053-20)
Supplement: FIG S2 [file mBio.01053-20-sf002.pdf]

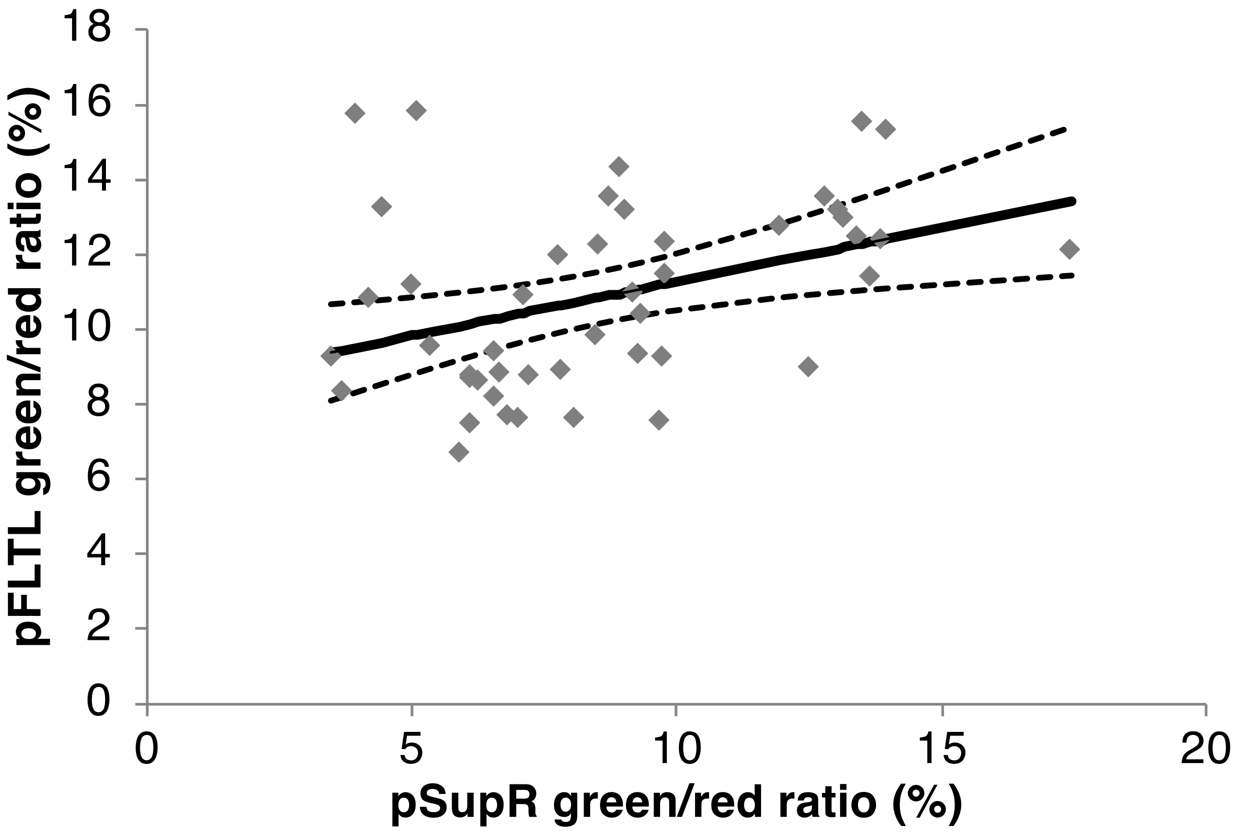

Figure S2: Comparison of the green to red fluorescence ratios in wild-type *Salmonella* carrying plasmid pFLTL or plasmid pSupR. Ratios of green fluorescence to red fluorescence produced by wild-type *S. enterica* serovar Typhimurium (14028s) harboring plasmid pFLTL, in which the constitutive *ffh* promoter drives transcription of a promoterless *gfp* gene and (separately) of a promoterless *dtomato* gene, or plasmid pSupR, in which the supercoiling-responsive *ydeJ* promoter drives transcription of a promoterless *gfp* gene and the constitutive *ffh* promoter drives transcription of a promoterless *dtomato* gene. Bacteria were grown in the following media: HH, HH800, HH800 pH 4.6, and HH + novobiocin (25μg/ml). Data are represented as individual points (gray squares), fit to a linear model (black line), and 95% confidence interval for the fit (dotted lines).
