# Supplementary material for: FEDS: a Novel Fluorescence-Based High-Throughput Method for Measuring DNA Supercoiling In Vivo
Source: mBio. 2020 Jul 28;11(4):e01053-20. doi: 10.1128/mBio.01053-20 (PMC7387798; doi:10.1128/mBio.01053-20)
Supplement: FIG S3 [file mBio.01053-20-sf003.pdf]

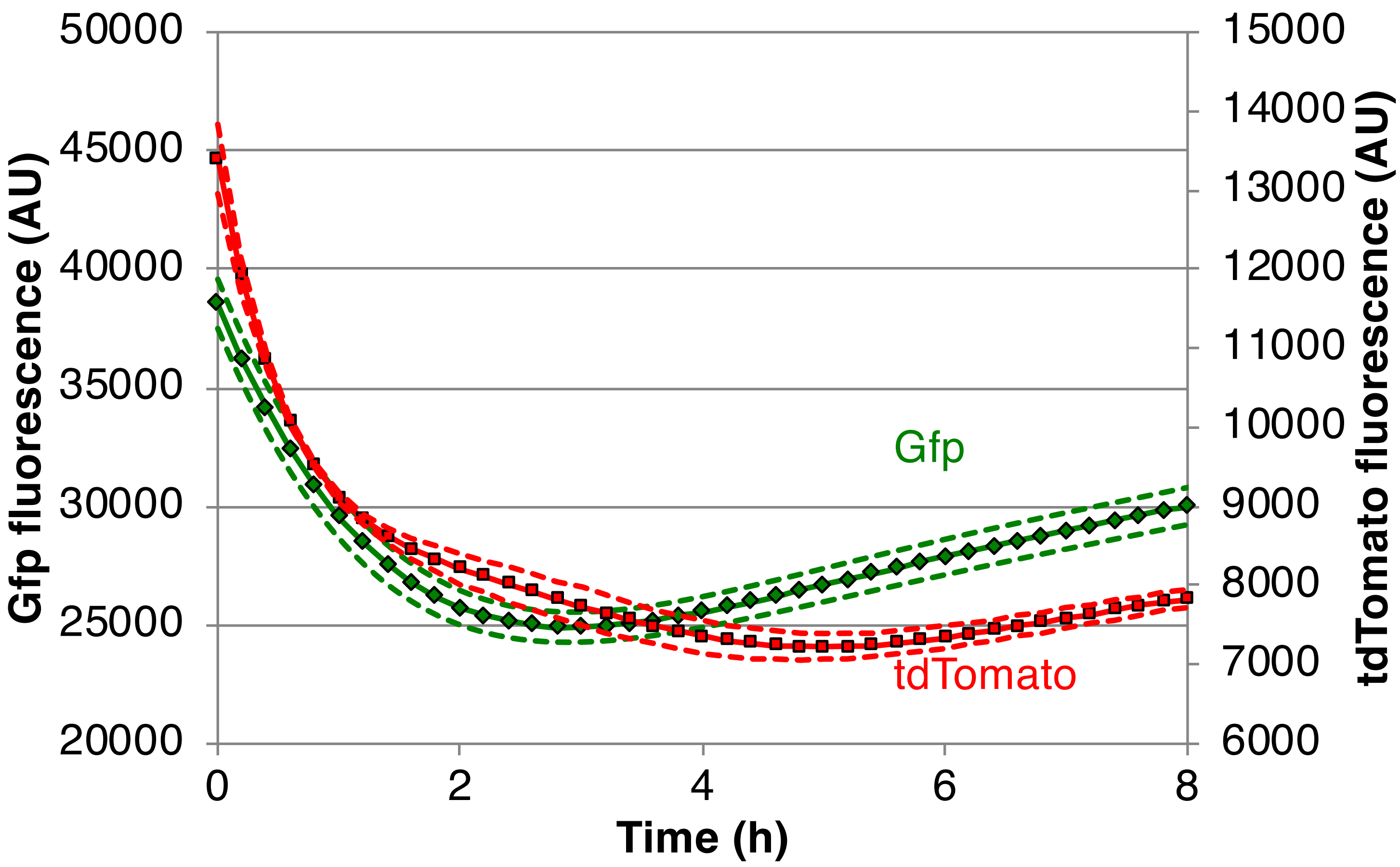

Figure S3: Raw green and red fluorescence measured in relation with Figure 5. *E. coli* MG1655/pSupR was grown in LB in 96-well plates. Data is represented as mean (solid lines) ± SD (dashed lines) of 3 replicates.
