# Supplementary material for: FEDS: a Novel Fluorescence-Based High-Throughput Method for Measuring DNA Supercoiling In Vivo
Source: mBio. 2020 Jul 28;11(4):e01053-20. doi: 10.1128/mBio.01053-20 (PMC7387798; doi:10.1128/mBio.01053-20)
Supplement: TEXT S1 [file mBio.01053-20-s0001.docx]

**Expanding FEDS to more distant bacterial species**

When considering the use of FEDS in a new bacterial species, pSupR should be validated to detect potentially unexpected regulation and to obtain a robust calibration curve. That is, the calibration obtained in one species cannot be extrapolated to another species because similar DNA supercoiling (as estimated by the classical agarose/chloroquine gel method) can result in different green-to-red ratios in the two species.

There are inherent advantages and disadvantages in pSupR specifying stable fluorescent proteins. On the one hand, stable fluorescent proteins confer more sensitivity than unstable ones. On the other hand, it gives pSupR-containing bacteria an intrinsic inertia because fluorescence decreases only due to dilution during growth as degradation of fluorescent proteins is exceedingly slow. Therefore, rapid effects on DNA supercoiling, such as those taking place immediately upon addition of the DNA gyrase inhibitor novobiocin (1), would require the use of a pSupR derivative in which the *gfp* and *tdtomato* genes are swapped by derivatives specifying unstable fluorescent proteins.

pSupR operates in organisms that recognize the promoter elements of enterobacterial species; however, pSupR would need to be modified for use in more distant organisms. The availability of an engineered *gfp* gene exhibiting reduced susceptibility to inhibition by H-NS and improved translation may improve GFP detection under some conditions (2). In addition, the fluorescence from Gfp or tdTomato is oxygen-dependent. Therefore, to measure the effects of anaerobiosis on DNA supercoiling (3), pSupR would need to be modified with genes specifying flavin-dependent fluorescent proteins (4), with the caveat that they tend to be less bright than oxygen-dependent fluorescent proteins.

**References**

1. Peter BJ, Arsuaga J, Breier AM, Khodursky AB, Brown PO, Cozzarelli NR. 2004. Genomic transcriptional response to loss of chromosomal supercoiling in Escherichia coli. Genome Biol 5:R87.

2. Corcoran CP, Cameron AD, Dorman CJ. 2010. H-NS silences gfp, the green fluorescent protein gene: gfpTCD is a genetically Remastered gfp gene with reduced susceptibility to H-NS-mediated transcription silencing and with enhanced translation. J Bacteriol 192:4790–4793.

3. Dorman CJ, Barr GC, Bhriain NN, Higgins CF. 1988. DNA supercoiling and the anaerobic and growth phase regulation of tonB gene expression. J Bacteriol 170:2816–2826.

4. Drepper T, Eggert T, Circolone F, Heck A, Krauß U, Guterl J-K, Wendorff M, Losi A, Gärtner W, Jaeger K-E. 2007. Reporter proteins for *in vivo* fluorescence without oxygen. Nat Biotechnol 25:443–445.
