# Supplementary material for: FEDS: a Novel Fluorescence-Based High-Throughput Method for Measuring DNA Supercoiling In Vivo
Source: mBio. 2020 Jul 28;11(4):e01053-20. doi: 10.1128/mBio.01053-20 (PMC7387798; doi:10.1128/mBio.01053-20)
Supplement: FIG S4 [file mBio.01053-20-sf004.pdf]

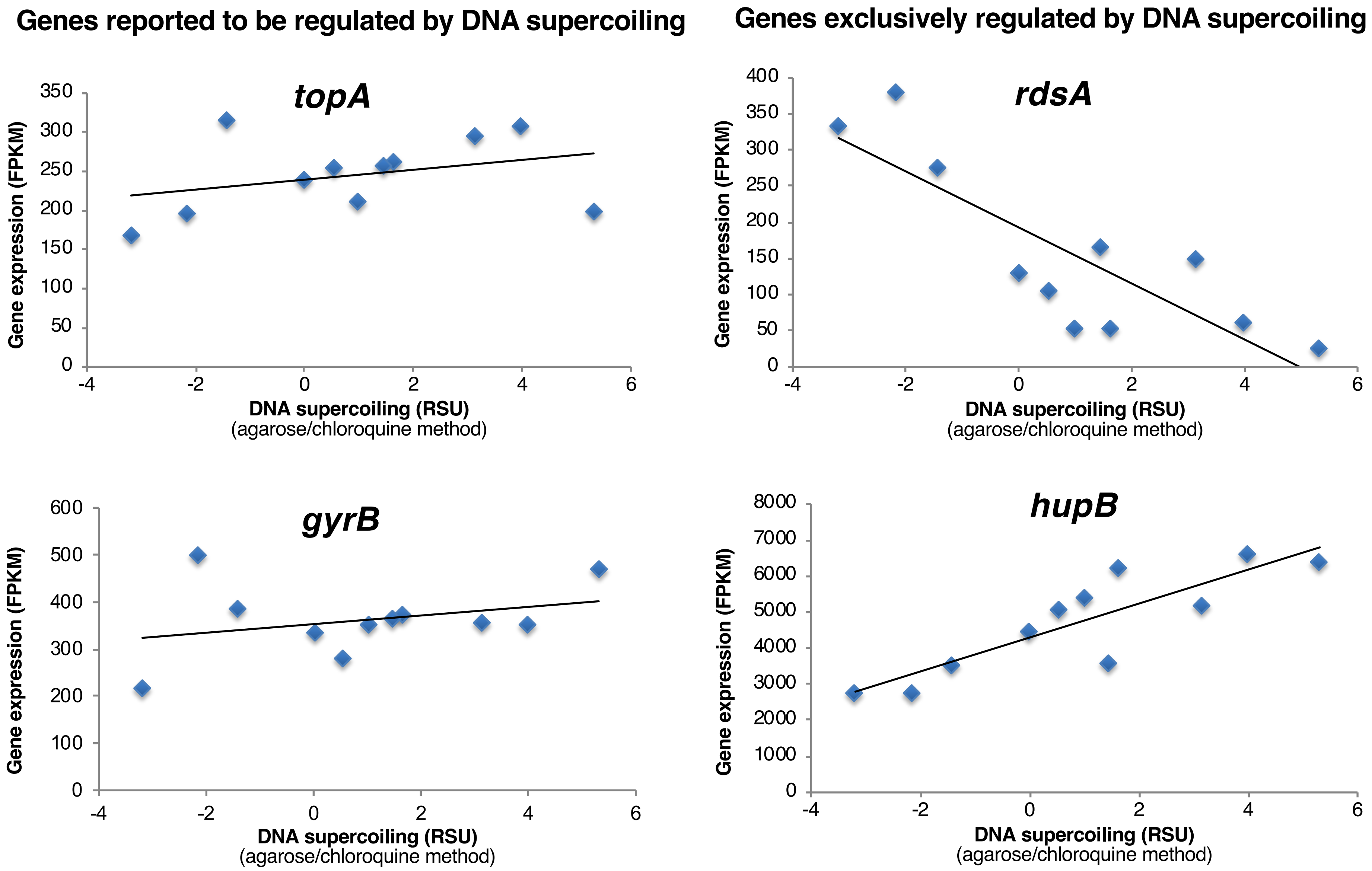

Figure S4: Expression of the *topA*, *gyrB*, *rdsA* and *hupB* genes as a function of conditions that alter DNA supercoiling.

DNA supercoiling was measured using the classical agarose/chloroquine gel method. Gene expression was evaluated by RNA-seq. Data for all *Salmonella* genes is presented in Supplementary File 2.
