# Supplementary material for: FEDS: a Novel Fluorescence-Based High-Throughput Method for Measuring DNA Supercoiling In Vivo
Source: mBio. 2020 Jul 28;11(4):e01053-20. doi: 10.1128/mBio.01053-20 (PMC7387798; doi:10.1128/mBio.01053-20)
Supplement: FIG S5 [file mBio.01053-20-sf005.pdf]

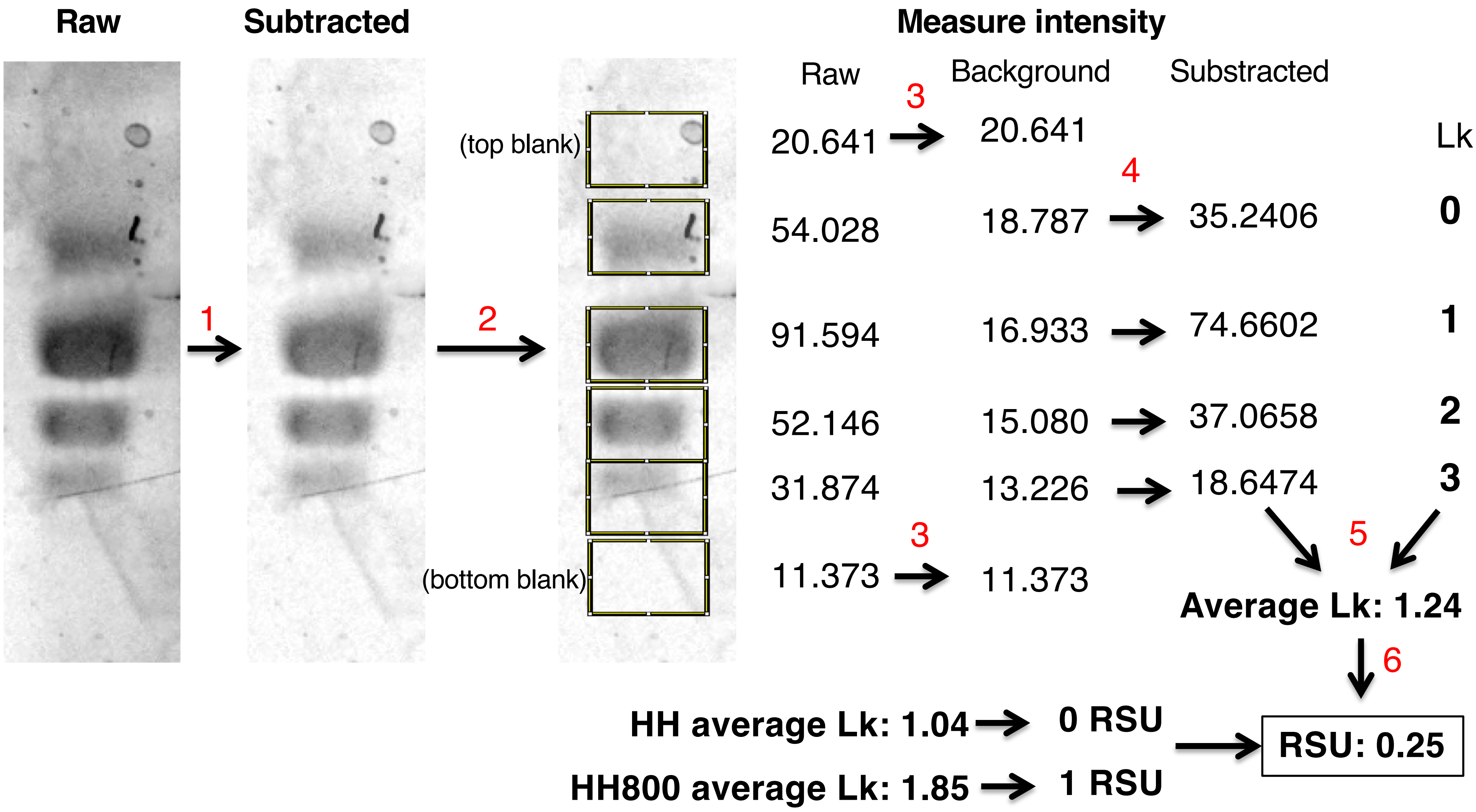

Figure S5: Detailed graphical example of calculation of RSUs from a gel image.

Please see full legend in next page.

Figure S5: Detailed graphical example of calculation of RSUs from a gel image.

1 (optional): If background has strong nonlinear variations, use the “substract background” function in ImageJ.

2: In ImageJ, draw a measurement area the size of the biggest band. Then, without changing it, measure the mean intensity of each band (“Measure” function). Depending on the image capture software and file format, white may have value 0 (like this example) or 255. This does not impact the rest of the analysis.

3: Calculate the background for subtraction by interpolating the top and bottom blanks linearly.

4: Subtract background from raw intensities.

5: Assign Lk (Linking number) values to each band (by arbitrary convention, the top one should be 0, but it has no impact on results). Then, calculate the average Lk weighted by the subtracted intensities.

6 (For *in vivo* experiments only): Take the calculated Lk from wild-type *Salmonella* grown in HH or HH800 media at 37°C to OD_{600}=0.8 loaded on the same gel. Linearly transform the Lk into RSU such that HH has a value of 0 RSU, and HH800 has a value of 1 RSU.

The “raw” gel image presented here is an edited composite image used for demonstration purposes only. Real gels may show up to 6 bands per 1 kb plasmid size.
