# Supplementary material for: FEDS: a Novel Fluorescence-Based High-Throughput Method for Measuring DNA Supercoiling In Vivo
Source: mBio. 2020 Jul 28;11(4):e01053-20. doi: 10.1128/mBio.01053-20 (PMC7387798; doi:10.1128/mBio.01053-20)
Supplement: FIG S6 [file mBio.01053-20-sf006.pdf]

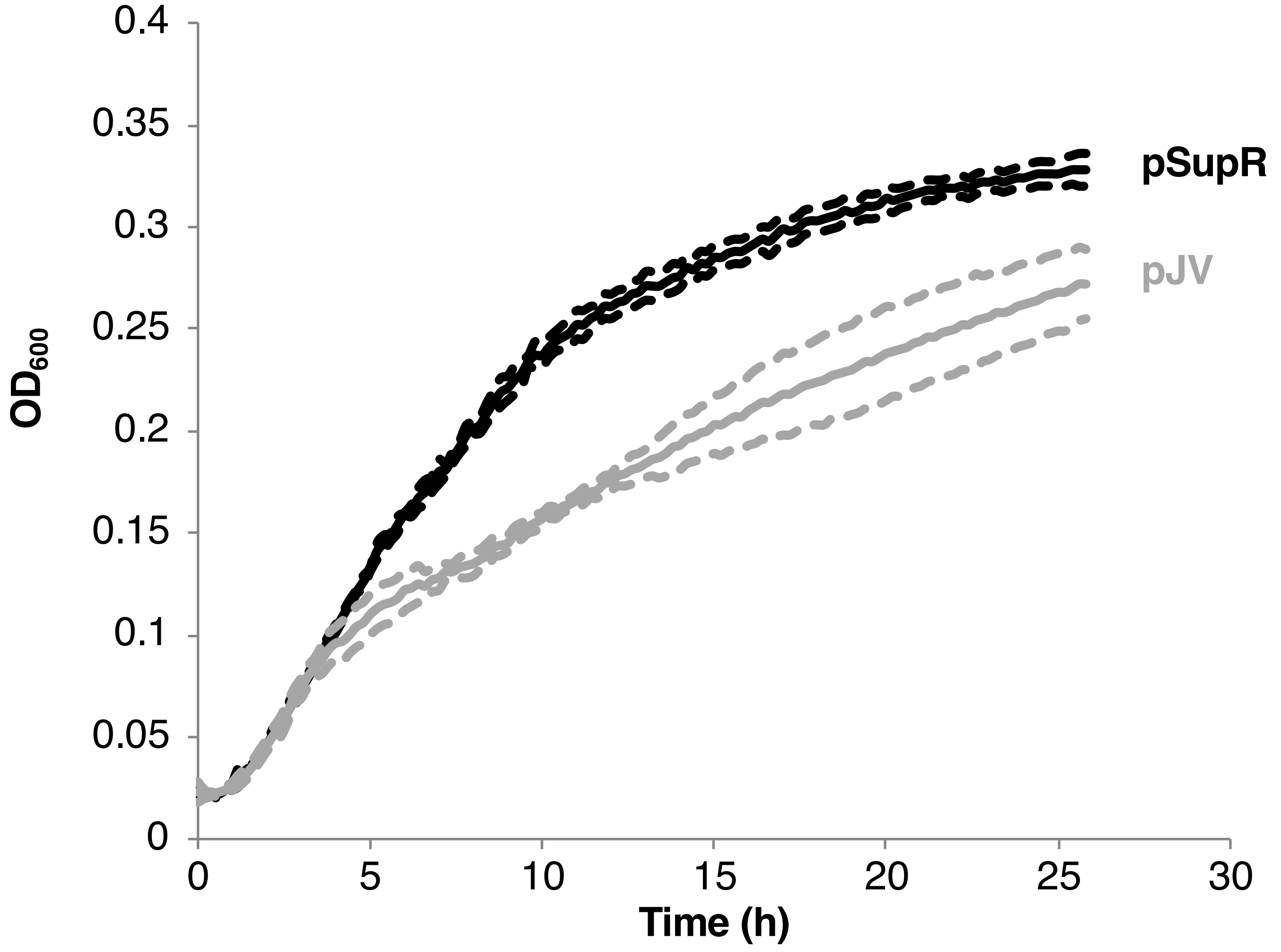

Figure S6: Growth of wild-type *Salmonella* bearing plasmid pSupR or plasmid pJV.

OD_{600} of Wild-type *S. enterica* serovar Typhimurium (14028s) harboring plasmid pSupR or plasmid pJV grown in HH800 medium in 96-well plates. Data are represented as mean (solid lines) ± SD (dashed lines) of 3 replicates.
